# Fine mapping of a candidate gene for cool-temperature-induced albinism in ornamental kale

**DOI:** 10.1186/s12870-020-02657-0

**Published:** 2020-10-07

**Authors:** Chenghuan Yan, Liying Peng, Lei Zhang, Zhengming Qiu

**Affiliations:** 1 Key Laboratory of Horticultural Plant Biology, Ministry of Education, College of Horticulture and Forestry Sciences, Huazhong Agricultural University, Wuhan, 430070 People’s Republic of China; 2 Hubei Key Laboratory of Vegetable Germplasm Enhancement and Genetic Improvement, Institute of Economic Crops, Hubei Academy of Agricultural Sciences, Wuhan, 430064 People’s Republic of China

**Keywords:** Albino trait, Semi-dominant inheritance, BSR-seq, Cytochrome P450 gene, Ornamental kale

## Abstract

**Background:**

The symptoms of cool-temperature-induced chlorosis (CTIC) are widely existed in higher plants. Although many studies have shown that the genetic mechanism of CTIC is generally controlled by recessive genes in model plants, the dominant inheritance of albinism has not been reported thus far. Here, two CTIC mutants, Red Kamome and White Kamome, were utilized to analyse the inheritance of the albino trait in ornamental kale. The objective of this investigation is to fine-map the target locus and identify the most likely candidate genes for albinism.

**Results:**

Genetic analysis revealed that the albinism in the inner leaves of ornamental kale followed semi-dominant inheritance and was controlled by a single locus in two segregating populations. BSR-seq in combination with linkage analysis was employed to fine-map the causal gene, named *AK* (*Albino Kale*), to an approximate 60 kb interval on chromosome C03. Transcriptome data from two extreme pools indicated that the differentially expressed gene of *Bol015404*, which encodes a cytochrome P450 protein, was the candidate gene. The *Bol015404* gene was demonstrated to be upregulated in the albino leaves of ornamental kale by qPCR. Additionally, the critical temperature for the albinism was determined between 10 °C and 16 °C by gradient test.

**Conclusions:**

Using two independent segregating populations, the albino mutants were shown to be controlled by one semi-dominant gene, *AK*, in ornamental kale. The *Bol015404* gene was co-segregated with albinism phenotypes, suggesting this unknown function P450 gene as the most likely candidate gene. The albino trait appeared caused by the low temperatures rather than photoperiod. Our results lay a solid foundation on the genetic control of albinism in ornamental kale.

## Background

Chlorophyll biosynthesis is the most important biochemical process on our planet [1]. The chlorophyll biosynthetic pathway occurs in chloroplasts, and involves many enzyme-catalysed reactions [2]. Therefore, chloroplasts are unique units of photosynthesis in green plants that generate multiple metabolic products of the photosynthetic processes [3]. Numerous studies have reported that chloroplasts are derived from proplastids, and the formation of chloroplasts involves the regulation of plastid and nuclear genes [4]. To date, substantial evidences have revealed that the biogenesis of chloroplasts is precisely regulated by a series of genes.

Low temperature is a common abiotic stress for most *Brassica* plants and is necessary for vernalization and reproduction. The symptoms of cool-temperature-induced chlorosis (CTIC) are usually observed in higher plants, such as Arabidopsis [5], rice [6, 7], maize [8], etc. Using genetic analysis approaches, many temperature-sensitive mutants have been identified in rice, including *tsc-1* [9], *cde1* [10], *ysa* [11], *tcd5* [12], etc. In mung beans, etiolated seedlings in the dark were completely repressed at 10 °C and were unable to turn green again under normal light conditions [13]. Thus, both dicots and monocots have CTIC symptoms, which are universal phenomena in higher plants. However, the molecular mechanisms of cool-temperature-induced chloroplast deficiency have not been fully elucidated.

Ornamental kale (*Brassica oleracea var. acephala*) and its related varieties, including curly kale (*B. oleracea* var. *sabellica* L.), thousand-head kale (*B. oleracea* var. *ramosa* DC.), marrow-stem kale (*B. oleracea* var. *medullosa* Thellg.), etc., belong to the members of kale of *B. oleracea* [14]. For human purposes, kales are divided into two main types: edible kale, which is used as a vegetable or fodder crop (such as curly kale), and ornamental kale, which is used in landscaping. Ornamental kale is one of the most popular ornamental crop worldwide. In China, ornamental kale is generally used as a landscape plant in winter due to its colourful morphology. The main colours of the cultivated ornamental kales are white and red. The investigation of CTIC symptoms in kale can be traced back to the middle of the twentieth century. In 1959, Martin discovered a dominant gene that likely controlled the albinism in winter, but a contradictory conclusion was made in summer [15]. Another study showed that the albino trait in ornamental kale is related to a chlorophyll deficiency in the inner leaves [16]. In a previous study, a red kale called Red Kamome was discovered to retain two independent traits for both anthocyanin accumulation and albinism, revealing that the albino phenotypes may be controlled by a different locus [17]. However, the genetic and molecular mechanisms of albinism remain poorly understanding in kale.

Albinism is a unique variation in kale that can be produced by undiscovered and infrequent genetic mechanisms. To confirm this hypothesis, the inheritance of this trait was carefully analysed using two independent segregating populations. This research aimed to elucidate this genetic relationship for further identification of the candidate gene. In this study, a rare semi-dominant inheritance pattern was repeatedly identified for albinism in ornamental kale. Furthermore, the target trait was fine-mapped within a narrow interval using BSR-seq and linkage analysis. Our study sheds light on the genetic mechanism controlling albinism in kale, and provides useful information for the further functional characterization of the candidate gene.

## Results

### Genetic analysis of the albino trait in ornamental kale

**Fig. 1:**
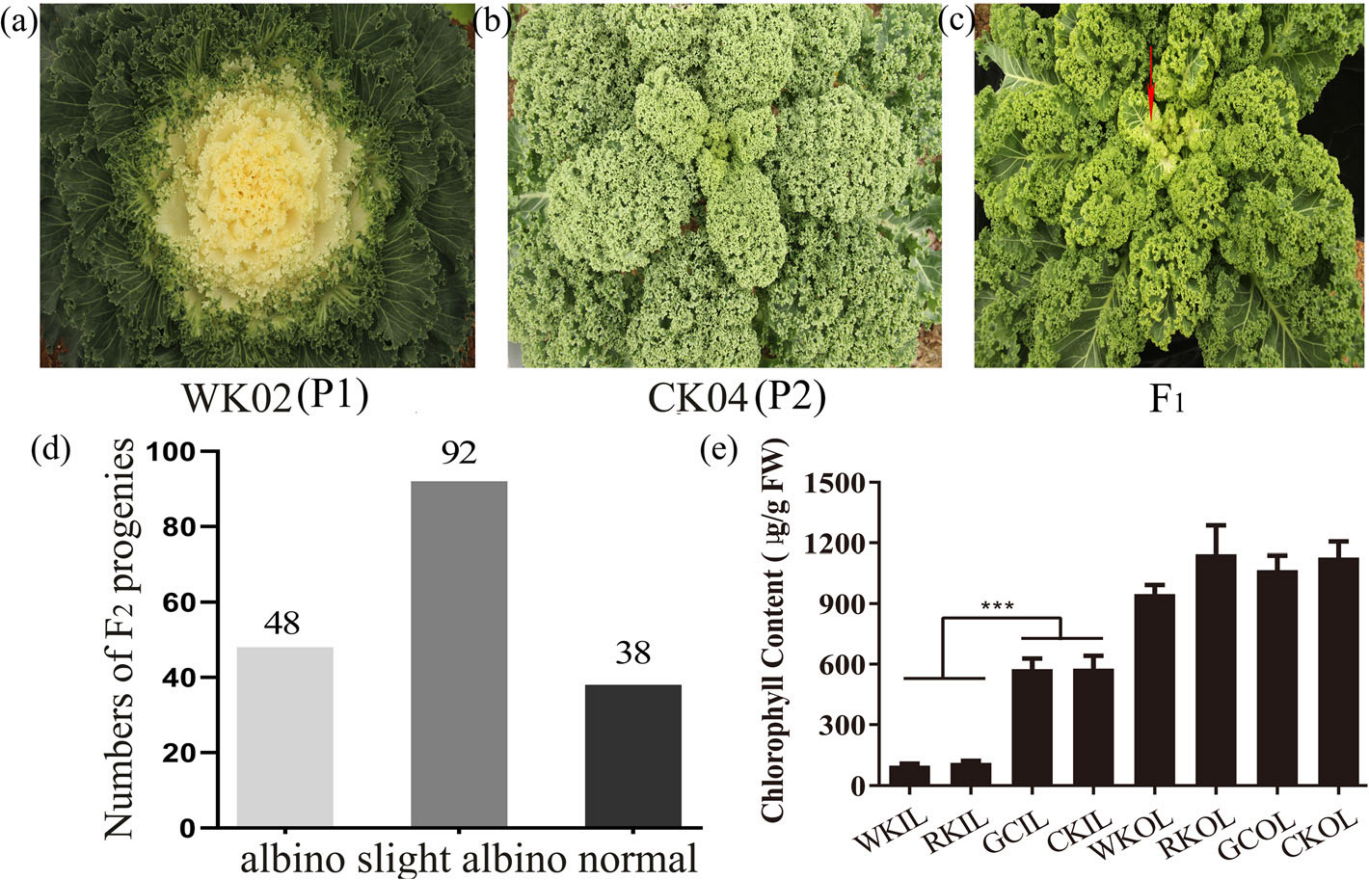
The phenotypes and chlorophyll contents of the parental lines and their progenies. **a** WK02. **b** CK04 (wild type). **c** The phenotype of F_1_ progeny. **d** The phenotypic distribution of F_2_ progenies. **e** Chlorophyll contents in WK02, RK01, green cabbage and CK04. RK, RK01; GC, green cabbage; WK, WK02; CK, CK04; OL, outer leaves; IL, inner leaves. *** means *p* < 0.001 by student's *t* test

Two albino mutants, RK01 and WK02, were utilized to analyse the inheritance of albinism in ornamental kale. A F_2_ segregating population was generated by WK02 (Fig. 1a) with albino phenotype in the inner leaves and CK04 (Fig. 1b) with green leaves, which F_1_ progenies showed slight albino phenotype in the inner leaves (Fig. 1c). The F_2_ progenies exhibited three leaf colour phenotypes, including 48 albino plants, 92 slight albino plants and 38 normal plants, with a segregation ratio of 1:2:1 (*χ*^2^ = 1.33, *P* = 0.52 > 0.05, Fig. 1d). The result indicates that the albino trait is controlled by a semi-dominant locus, named *Albino Kale* (*AK*), in WK02. Additionally, the albino phenotype was also discovered in the F_1_ plants and BC_1_ population of RK01 and green cabbage [17]. A total of 603 BC_1_ progenies were used to analyse the inheritance of the albinism independently. Phenotyping of 603 progenies revealed that the segregation ratio of 304 albino individuals and 299 green individuals were 1:1 (*χ*^2^ = 0.04, *P* = 0.84 > 0.05, Figure S1 and Table 1). Furthermore, a small BC_1_F_2_ population was selected to further determine the genetic relationship. Progenies of the BC_1_F_2_ population showed three leaf colour phenotypes, including 27 albino plants, 52 slight albino plants and 20 normal plants, with Mendelian ratio of 1:2:1 (*χ*^2^ = 1.24, *P* = 0.54 > 0.05, Figure S2 and Table 1). These results also indicate that the albinism of ornamental kale is a unique semi-dominant trait, which is probably controlled by single gene. Chlorophyll contents of parental lines were measured at four-month-old stage. Chlorophyll contents of the albino region in RK01 and WK02 were significantly lower than that of the inner leaves in green cabbage and CK04 (*P* < 0.001), while it had no significant difference in the outer leaves among RK01, WK02, CK04 and green cabbage (Fig. 1e). The findings suggested that the albino phenotype might be caused by repressing the chlorophyll biosynthesis and/or chloroplast development in ornamental kale.

**Table 1:** The Chi-square (*χ*^2^) of leaf colour segregation in the BC_1_ and its derivative populations

Population	Plant numbers	No. albino individuals	No. slight albino individuals	No. normal individuals	Expected ratio	***χ***^**2**^
F_1_	21	–	21	0	–	–
BC_1_	603	–	304	299	1:1	0.04^a^
BC_1_F_2_	99	27	52	20	1:2:1	1.24^a^

^a^χ^2^ > χ^2^_0.05_ = 3.84 were regarded as significant difference

### Phenotyping and chloroplast analysis in albino kale genotypes

**Fig. 2:**
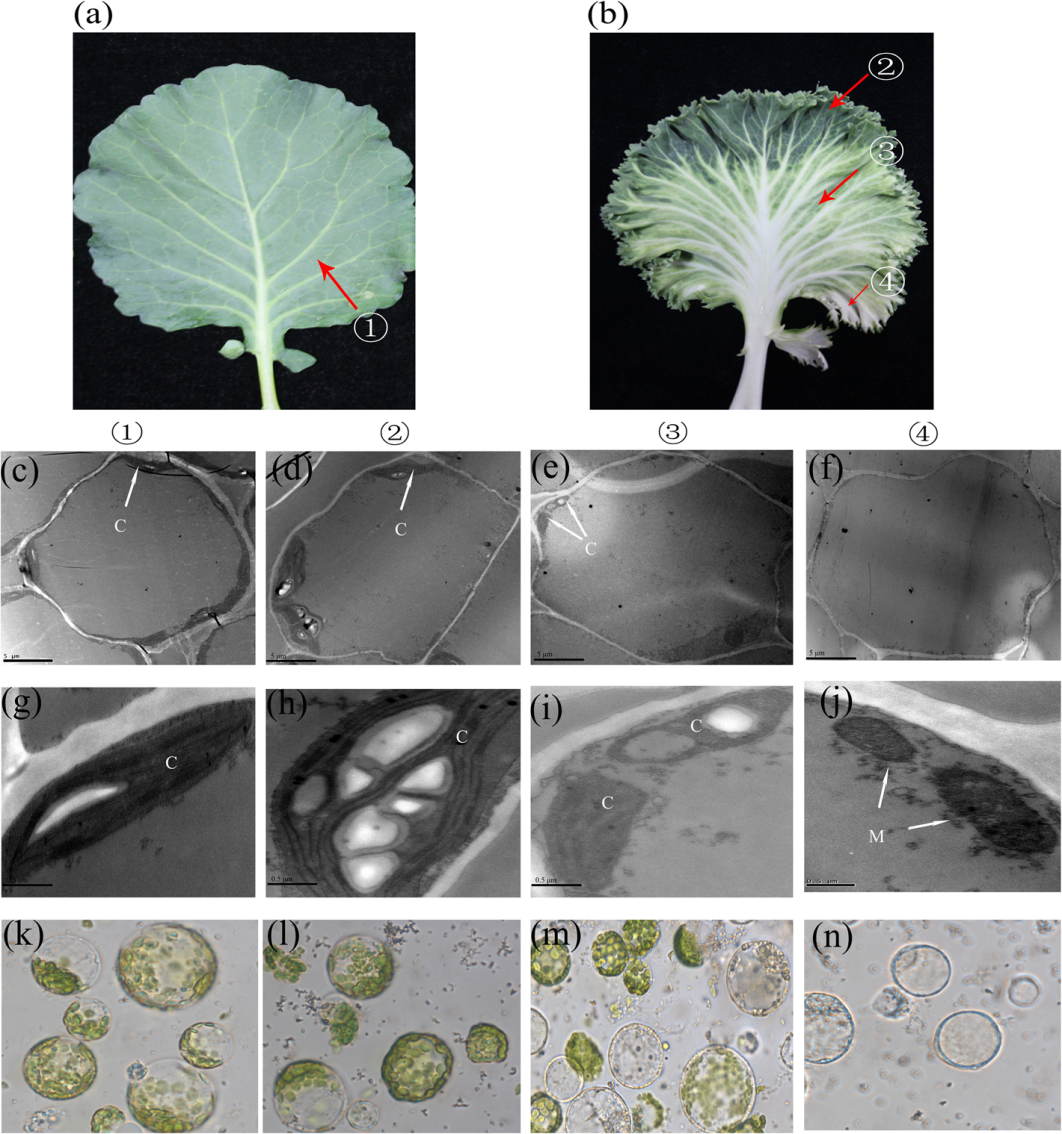
Cytologic characteristics of green cabbage and WK02. **a** Leaf of green cabbage. **b** Leaf of WK02. Transmission electron microscope images of position ① shown in **c** and **g**, position ② shown in **d** and **h**, position ③ shown in **e** and **i**, position ④ shown in **f** and **j**. Protoplast images (60x) of position ①-④ shown in **k**-**n**, respectively. The bars of **c**-**f** represent 5 μm; The bars of **g**-**j** represent 0.5 μm. C, chloroplast; M, mitochondria

To understand how chlorophyll and/or chloroplast cause albino phenotypes, transmission electron microscopy was ultilized to observe the ultrastructures of the chloroplasts in WK02 and green cabbage. Three regions in the leaves of WK02, including all-green, albino-green and all-albino tissues, were selected for the observation of their chloroplast morphology (Fig. 2b), and green cabbage was used as a control (Fig. 2a). Unlike in green cabbage, the number of chloroplasts gradually decreased to zero from the all-green to the all-albino tissues in WK02 (Fig. 2c-f). In the all-green tissues of WK02, the chloroplast morphology showed normal development at low temperature (Fig. 2h). In addition, the chloroplast ultrastructure revealed a loose arrangement and abnormal grana stacks, and some vacuoles were even detected in the albino-green tissues (Fig. 2i). Interestingly, chloroplasts were not observed in the all-albino tissues (Fig. 2f), but mitochondria were (Fig. 2j). These results were consistent with the phenotype and chlorophyll content in WK02. Therefore, we inferred that no chloroplasts were transformed from proplastids in the all-albino tissues of WK02. To further confirm this hypothesis, protoplasts of WK02 and green cabbage were isolated and observed. Similar to those of the green cabbage, the protoplasts of all-green tissues had normal chloroplast morphology in WK02 (Fig. 2k-l), and no chloroplasts were identified in the protoplasts of all-albino tissues (Fig. 2n). In the green-albino tissues, the morphology of both normal chloroplast and chloroplast-free was clearly observed, revealing an intermediate state between the all-green and all-albino tissues (Fig. 2m). These results indicate that the albino phenotype in the inner leaves of White Kamome was caused by chloroplast deficiency.

### *AK* gene mapping

**Fig. 3:**
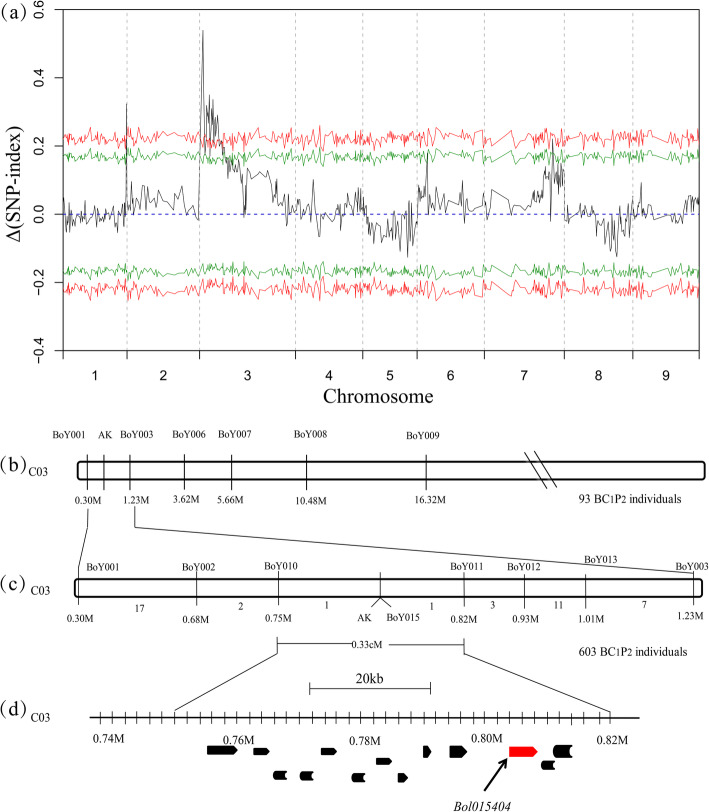
Mapping of the *AK* gene in the BC_1_ population **a** The average Δ (SNP-index) graph based on the data of N-pool and A-pool against reference genome 02–12 (*X*-axis). Peak of target region was shown on Chromosome C03. The CIs were revealed with green lines (*P* < 0.05) and red lines (*P* < 0.01). **b** The *AK* gene was mapped between molecular markers BoY001 and BoY003 using 93 randomly selected BC_1_ individuals. **c** Fine mapping the *AK* gene between molecular markers BoY010 (754, 756 bp) and BoY011 (815, 202 bp). The numbers below chromosome indicate the number of recombinants between two markers. **d** Schematic diagram of predicted genes in the *AK* locus. The broad arrows represent predicted thirteen genes in the candidate region. The *Bol015404* was considered a candidate gene

BSR-seq was employed to preliminarily map the *AK* gene. Two pools, A-pool and N-pool, generated 5.29 Gb and 6.21 Gb of clean data by RNA-seq, respectively. The Δ (SNP-index) graph was calculated based on the data of N-pool and A-pool against the reference genome 02–12 [18]. A peak for *AK* locus occurred in the front of chromosome C03 (Fig. 3a), indicating a single locus controlling the albinism in ornamental kale.

At CI values of 95 and 99%, the *AK* gene was anchored in 0–15.10 (*P* < 0.05) Mb and 0–13.30 Mb (*P* < 0.01) on chromosome C03 (Fig. 3a). Furthermore, we screened 93 randomly selected progenies of the BC_1_ population using six molecular markers, including BoY001, BoY003, BoY006, BoY007, BoY008 and BoY009. Using the 93 progenies, the *AK* gene was preliminary mapped between molecular markers BoY001 (300, 692 bp) and BoY003 (1, 229, 918 bp) (Fig. 3b).

To fine-map the *AK* gene, we screened 603 progenies of the BC_1_ population using molecular markers BoY001 and BoY003, with identifying twenty and twenty-two recombinants, respectively. New polymorphic markers were developed between BoY001 and BoY003, including BoY002, BoY010, BoY011, BoY012, BoY013 and BoY015. Finally, *AK* gene was delimited in the region between molecular markers BoY010 (754, 756 bp) and BoY011 (815, 202 bp), with a physical region of approximate 60 kb and genetic distance of 0.33 cM (Fig. 3c).

### Candidate *AK* gene prediction

In the target region, thirteen predicted genes were obtained between BoY010 and BoY011 (Table 2, Fig. 3d). Then, FGENESH and GENESCAN were operated to identify thirteen ORFs, which were consistent with the reference genome. Five predicted genes, including *Bol015395*, *Bol015396*, *Bol015399*, *Bol015401* and *Bol015402*, encode uncharacterized proteins. *Bol015394* is likely to encode a clathrin adaptor complex subunit protein that shares 78.1% amino acid identity with AT5G05010. *Bol015397* encodes ubiquitin-conjugating enzyme 22, which is involved in female gametophyte development [19]. *Bol015398* is a MYBC1-like transcription factor that negatively regulates the freezing tolerance in Arabidopsis [20]. *Bol015406* encodes a cellulose synthase involved in the cellulose biosynthesis process [21]. *Bol015400*, *Bol015403* and *Bol015405* are related to fundamental biological processes, such as repressing cysteine proteinase, RNA polymerase transcription, and autophagosome assembly, respectively. *Bol015404* encodes a cytochrome P450 708A subfamily protein, which is orthologous with *AT3G44970*, sharing 73.7% amino acid identity. The function of Bol015404 has not yet been verified. In the fine-mapping region, only one gene of *Bol015404* was differentially expressed between the A-pool and N-pool (FPKM value of the A-pool/N-pool ≈ 27.1). Thus, the *Bol015404* gene was temporarily considered a candidate gene (Fig. 3d).

**Table 2:** The thirteen predicted genes between molecular markers BoY010 and BoY011

**Gene name**	**start**	**stop**	**Length (bp)**	**identifier**
Bol015394	756,300	763,338	2013	Clathrin adaptor complexes subunit protein
Bol015395	763,580	764,449	204	Uncharacterized protein
Bol015396	765,035	765,273	153	Uncharacterized protein
Bol015397	767,150	768,367	618	Ubiqutin-conjugating enzyme E2 22-like
Bol015398	775,158	775,907	750	Transcription factor MYBC1-like
Bol015399	778,895	780,322	951	Uncharacterized protein
Bol015400	780,861	781,878	687	Cysteine proteinase inhibitor 7
Bol015401	782,878	783,426	549	Uncharacterized protein
Bol015402	791,186	791,626	441	Uncharacterized protein
Bol015403	795,555	797,410	1191	Probable mediator of RNA polymerase II transcription subunit 26b
**Bol015404**	**805,055**	**808,102**	**1377**	**Cytochrome P450 708A family protein**
Bol015405	808,399	809,967	1119	Autophagy-related protein 18E
Bol015406	810,685	813,318	1884	Cellulose synthase A catalytic subunit 3

**Fig. 4:**
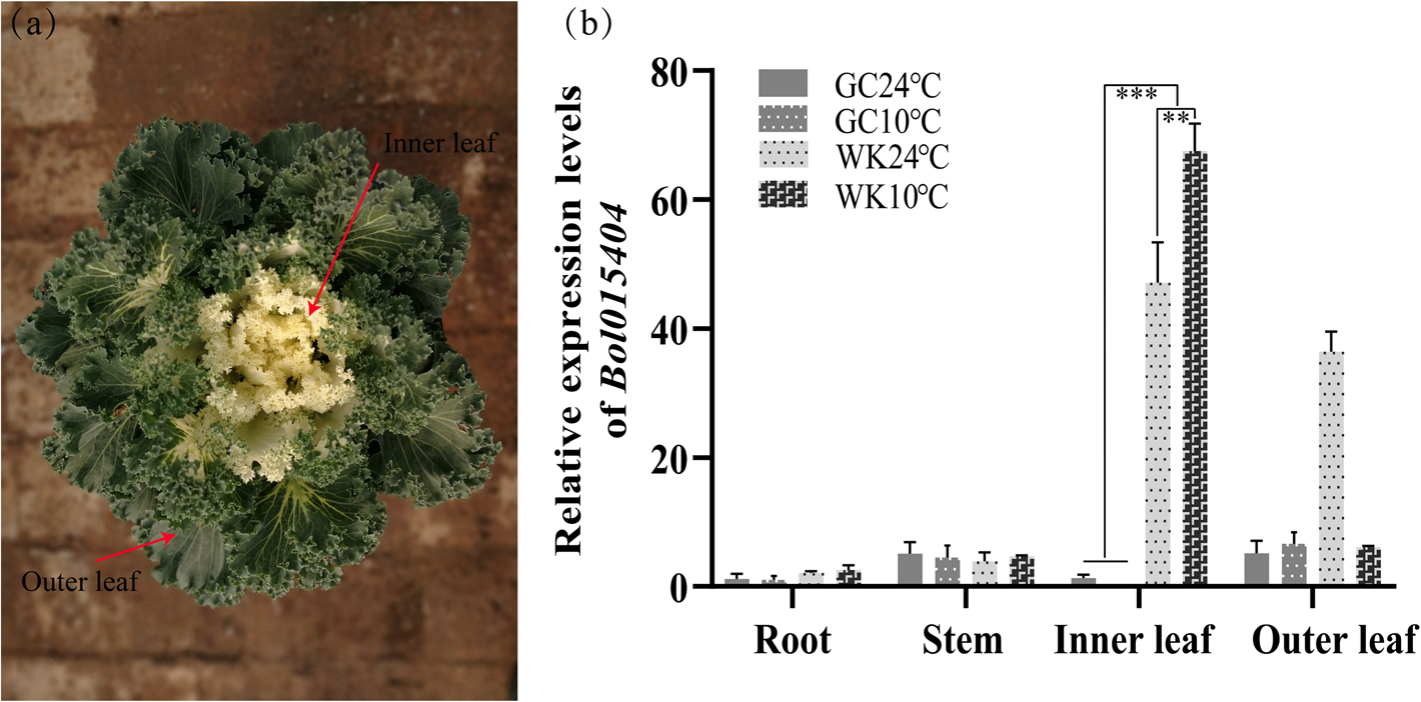
Relative expression levels of *Bol015404* (**a**) Phenotype of WK02 under low temperature. **b** Expression levels of *Bol015404* in different tissues at 10 °C and 24 °C. Data represent mean ± SD (*n* = 3). The expression level of *Bol015404* in the green cabbage at 24 °C was set as 1. WK, WK02; GC, green cabbage. ** and *** means *P* < 0.01 and *P* < 0.001 by student’s *t* test

To determine the possible candidate gene, the expression levels of *Bol015404* under different temperature treatments were detected by qPCR. The results revealed that the expression levels of *Bol015404* were relatively low in the root and stem tissues of WK02 and green cabbage (Fig. 4). However, *Bol015404* had higher expression in the inner leaves of WK02 than in those of green cabbage (*P* < 0.001) at 10 °C and 24 °C. Compared with those in the 24 °C treatment, the expression levels of *Bol015404* were significantly upregulated in the inner leaves of WK02 at 10 °C (*P* < 0.01). The findings indicate that the expression of *Bol015404* in the inner leaves may be induced by low temperature*.* Furthermore, a SNP marker, BoY015, located in the intron region of *Bol015404*, co-segregated with the phenotypes in the BC_1_ population (Fig. 3c). Thus, *Bol015404* was regarded as the most likely candidate gene regulating albinism.

### Sequence analysis of *Bol015404*

**Fig. 5:**
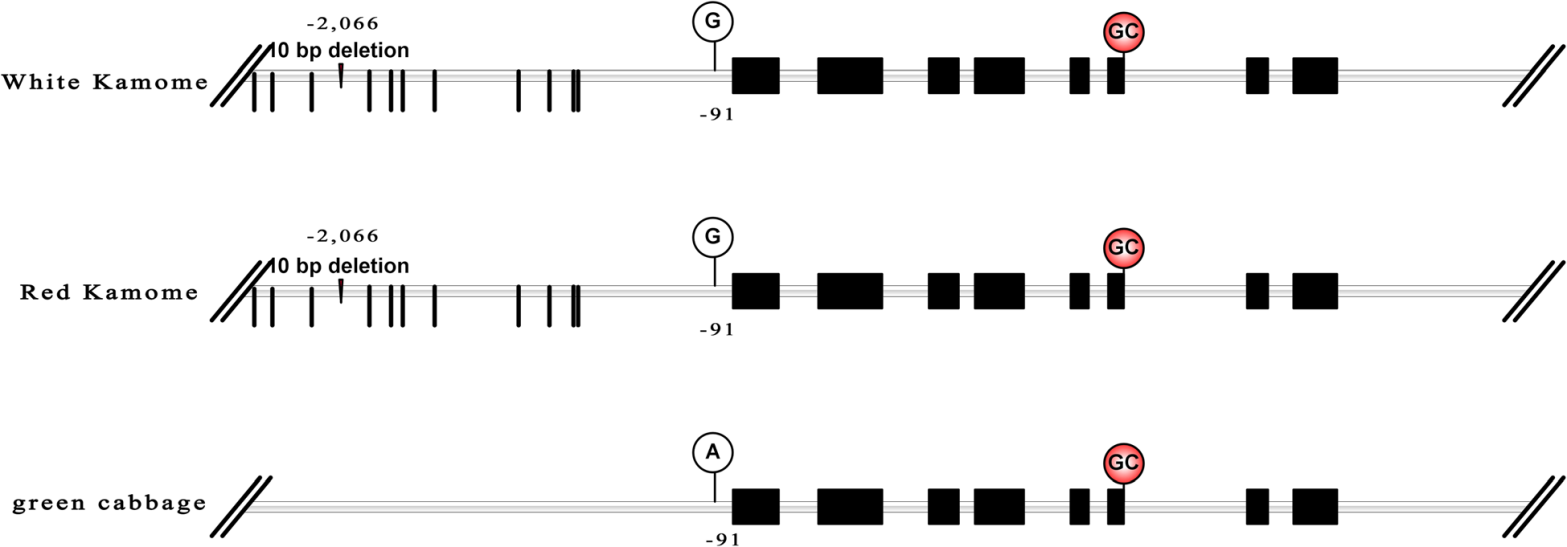
Schematic diagram of *Bol015404* alleles in WK02, RK01 and green cabbage

We amplified and sequenced the genomic sequences (including approximate 2.5 kb promoter region) of *Bol015404* in RK01, WK02 and green cabbage. The genomic sequences revealed complete identity in the intron and promoter regions between RK01 and WK02 (Text S1). In the coding regions, two albino kales, RK01 and WK02, and green cabbage had 100% sequence identity (Figure S3). Interestingly, the splice site of intron 6 was occasionally GC-AG rather than GT-AG in the three parental lines. We analysed the promoter sequences of WK02 and green cabbage in detail. Unlike in green cabbage, 12 point mutations were distributed in the promoter region of WK02, and a 10 bp deletion was discovered at the − 2066 bp location (Fig. 5). We inferred that these mutations in the promoter region might be responsible for the induced expression of *Bol015404* in the inner leaves of WK02*.*

### Albinism induced by low temperature rather than photoperiod

**Fig. 6:**
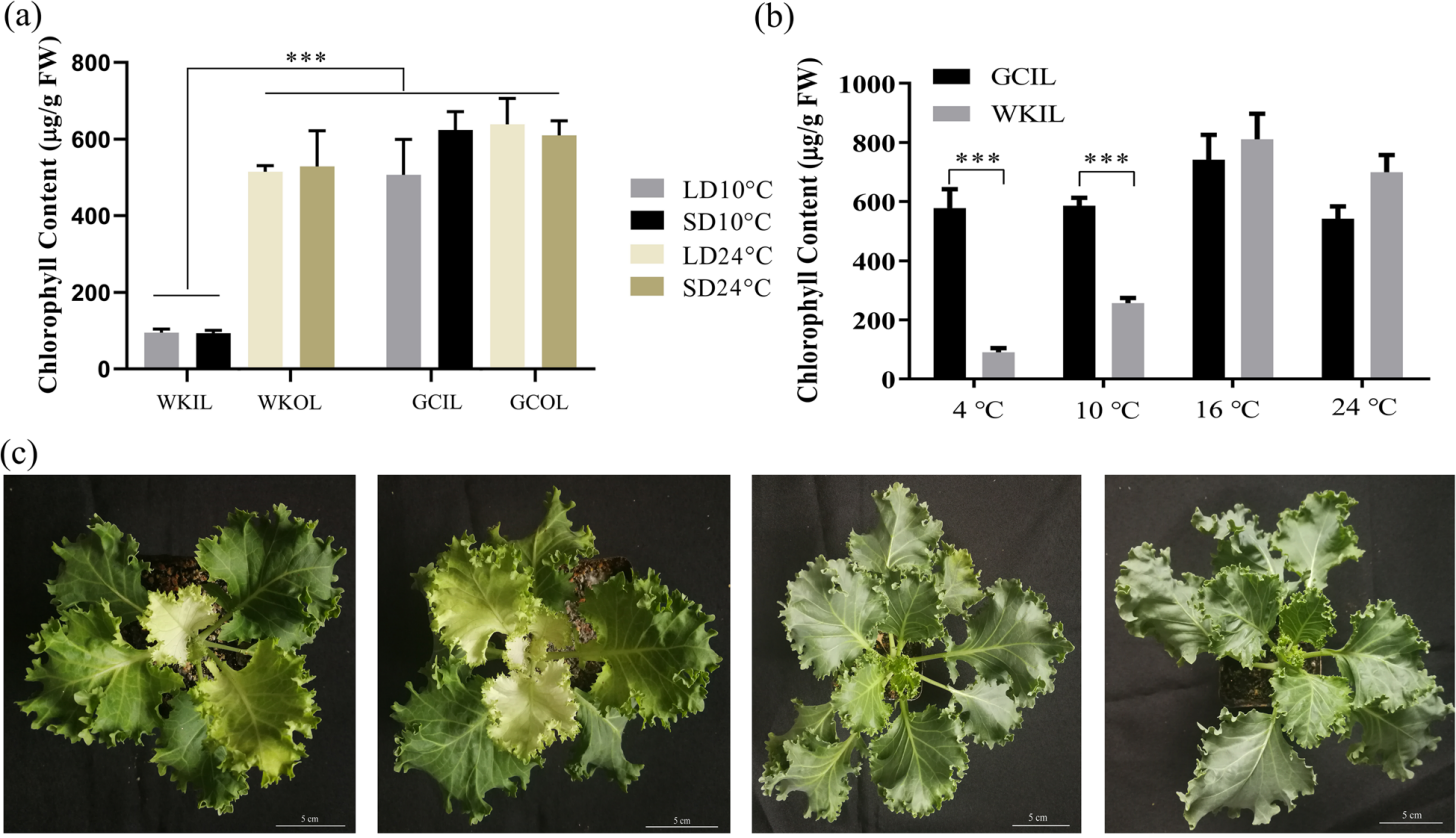
Chlorophyll contents and phenotypes under different photoperiods and temperatures **a** Chlorophyll contents of WK02 and green cabbage under different photoperiods (long-day and short-day) and temperatures (24 °C and 10 °C) treatments. **b** Chlorophyll contents of WK02 under different temperature treatments. **c** The phenotypes of WK02 under different temperature treatments including 4 °C, 10 °C, 16 °C and 24 °C from left to right. LD, long-day photoperiod; SD, short-day photoperiod; WK, WK02; GC, green cabbage; OL, outer leaves; IL, inner leaves. *** means *P* < 0.001 by student’s *t* test. Bars = 5 cm

The albino phenotype of ornamental kale was usually observed in autumn and winter fields. We speculated that the albinism was induced by low temperature and/or a short photoperiod. To determine the environmental factors, four temperature and photoperiod treatmeats were performed to explore the key abiotic factor. The LD and SD photoperiods did not cause significant differences in chlorophyll contents between WK02 and green cabbage. However, the phenotype of WK02 revealed obvious albinism in the inner leaves, and its chlorophyll content was significantly lower under the low temperature (10 °C) than under the normal temperature (24 °C) (Fig. 6a). Thus, the albino phenotype was induced by low temperature rather than by the photoperiod in ornamental kale.

To explore the critical temperature for albinism, a temperature gradient test was performed in this study. The results showed that the inner leaves of WK02 gradually developed the albinism at 4 °C and 10 °C, but it always exhibited a normal green colour in the inner leaves after three weeks at 16 °C and 24 °C (Fig. 6c). Therefore, the chlorophyll content of WK02 decreased significantly under low temperatures (4 °C and 10 °C) compared with other temperatures (*P* < 0.001, Fig. 6b).

## Discussion

In this study, bulked segregant analysis (BSA) in combination with RNA-seq (BSR-seq) [18] was employed to preliminarily map the target region of the albino phenotype in ornamental kale. A single peak was identified on chromosome C03 (Fig. 3a) by calculating the values of Δ (SNP-index) between two pools, the A-pool and the N-pool. These results demonstrated that BSR-seq was a powerful approach for identifying the corresponding region of the target trait. Additionally, BSA in combination with DNA resequencing (QTL-seq) can also promote the efficiency of genetic mapping [19]. Therefore, BSR-seq and QTL-seq have been widely used to identify several causal genes in *B. oleracea* species, such as *BoLl* [20, 21], *BoPs* [22], *BoMYB2* [17], *BoCCD4* [23, 24]. Unlike QTL-seq, BSR-seq takes advantage of differentially expressed genes from two pools, which simultaneously provides more information for candidate gene selection. Additionally, we mapped the albino trait using the BC_1_ segregating population (Table S1), which the population had been utilized to elucidate the genetic mechanism of anthocyanin accumulation in ornamental kale [17]. Thus, the efficiency of map-based cloning was doubled by constructing suitable population.

The molecular mechanisms of albinism formation are complex. They are related to multiple biochemical processes, such as chlorophyll biosynthesis [25], carotenoid biosynthesis [26], and heme metabolism [27]. Low temperatures are usually considered a major abiotic stress that causes albinism in plants. Low-temperature-induced chlorosis/albinism, also called CTIC symptoms, has been reported in many higher plants, including Arabidopsis [5] and rice [6, 7]*.* Generally, albino phenotypes are regarded as abnormal and negative traits in most plant breeding contexts [25]. However, the variegated and colourful leaves in ornamental kale are very popular among consumers because of their beautiful morphology [28]. Although a series of genes are related to chloroplast biogenesis at low temperatures, these mutants are usually controlled by recessive genes. In our study, two temperature-sensitive albino mutants, RK01 and WK02, were identified as showing a semi-dominant trait, rather than universal recessive inheritance. Using BSR-seq and linkage analysis, we narrowed the *AK* gene to an approximate 60 kb region on chromosome C03. However, these genes in the target region had neither chlorophyll biosynthesis or breakdown genes, such as *DVR* [29], *CHLG* [30], *CAO* [31], nor any well-known genes that regulate chloroplast biogenesis, such as the PPR family [11]. These results suggest that the albinism in ornamental kale is probably controlled by some unknown mechanisms.

Plant genomic sequencing revealed that the cytochrome P450 gene family is one of the largest gene superfamilies in higher plants. The number of P450s is estimated to be approximately 1% of all annotated genes [32]. For instance, 246 and 356 P450s were identified in Arabidopsis and rice, respectively [33]. The functions of P450 genes in plants involve various biochemical reactions and biosynthesis processes, such as those related to sterols [34], plant hormones [32], defence compounds [35] and leaf development [36]. Based on the promoter variations and differentially expressed genes, *Bol015404*, encoding a cytochrome P450 protein, was selected as the most likely candidate gene for *AK*. In this study, *Bol015404* was an uncharacterized gene belonging to the CYP708A subfamily. The expression of *Bol015404* was induced by a low temperature of 10 °C in the newly grown area. Therefore, upregulated expressions may lead to the development of albinism in the inner leaves of ornamental kale. Interestingly, *CYP708* genes are absent in rice but present in Arabidopsis, suggesting differences between monocots and dicots in the corresponding metabolites [33]. In the CYP708A subfamily, CYP708A2 has been characterized as being involved in triterpene synthesis by operon-like clusters in *Arabidopsis thaliana* [37]. However, the functions of other members of the CYP708A subfamily have not been validated in plants. Studies of the molecular mechanism of albinism in ornamental kale will broaden our knowledge of chloroplast development and biogenesis.

## Conclusions

Two albino mutants with semi-dominant inheritance displaying CTIC symptoms were discovered in ornamental kale. We identified the target region harbouring the candidate gene for albinism using the BSR-seq method. The *AK* gene was fine-mapped to a narrow region of 60 kb, with a genetic distance of 0.33 cM. Thirteen genes were predicted in the mapping region, and the cytochrome P450 gene *Bol015404* was selected as the most likely candidate gene for *AK* based on its differential expression and promoter variations. The effects of temperature and light treatments revealed that the low temperatures, rather than the photoperiod, were the key factor for inducing albinism in ornamental kale. Additionally, the critical temperature for the albinism of ornamental kale was determined between 10 °C and 16 °C by the gradient test. The present study provided a novel type of albinism in higher plants, it also laid a foundation for understanding the genetic control of this trait in ornamental kale; a candidate gene for *AK* was identified.

## Methods

### Plant materials and phenotypes

Two commercial varieties of ornamental kale (*B. oleracea* var. *acephala*) with albino phenotypes, Red Kamome and White Kamome (TAKII SEED, Japan), were used in this study. Two other materials, the green cabbage “HGDH” (*B. oleracea* var. *capitata*) and the curly kale “Zhou Ye Yu Yi” (Dongsheng Seeds, China), were utilized as normal parents for population construction. Green cabbage “HGDH” is a double haploid (DH) line, that was kindly provided by Professor Taotao Wang from Huazhong Agricultural University. All commercial materials were self-pollinated over three generations, confirming that the phenotypes were stable and consistent, especially those for leaf colour. Three parental lines derived from Red Kamome, White Kamome and “Zhou Ye Yu Yi” by self-pollinated were named as RK01, WK02 and CK04, respectively. A backcross segregating population was generated by RK01 and green cabbage “HGDH” in our previous study [17]. The BC_1_ population can be used to elucidate the genetic mechanism of anthocyanin accumulation and albinism in ornamental kale, because two traits are independently controlled by two loci (Table S1). A BC_1_F_2_ population was selected to further verify the genetic relationship of albino trait in ornamental kale. Additionally, an F_2_ population was generated by WK02 and CK04 to analyse the genetic relationship of albino traits between Red Kamome and White Kamome. In late August or early September, all segregating populations were planted at the “Huangtupo” base at Huazhong Agricultural University (Wuhan, China). Albino phenotypes were first observed in late October and early November. The average high temperature and low temperature were 30 °C and 20 °C in September 2014. However, the average high temperature and low temperature decreased to 14 °C and 7 °C in November 2014, respectively (information derived from www.tianqi.com). These phenotypes were identified twice at four-month-old plants through visual observation. Four temperature and photoperiod treatmeats were performed in an artificial climate chamber. The long-day (LD) and short-day (SD) photoperiods were implemented under 16 h/8 h (light/dark) and 8 h/16 h (light/dark), respectively. Fluorescent lamps were used as the light source for plant growth, and the light density was approximately 280 μmoles/m^2^/s. The relative humidity (RH) was set at 75%. To explore the critical temperature, a temperature gradient experiment was accomplished under the LD photoperiod with different temperatures, namely, 4 °C, 10 °C, 16 °C and 24 °C. These above treatments were performed on three-week-old plants, and their phenotypes were identified after three weeks.

### Chlorophyll measurement

Fresh leaves (0.2 g) were snipped into small pieces excluding vein and petiole. Chlorophyll was extracted with 10 mL ethanol solution (96%, v/v). The concentration of chlorophyll was measured at 649 nm and 665 nm [38]. In this study, chlorophyll contents of the parental lines were detected using four-month-old plants, and other treatment materials were measured at corresponding growth stages. All measurements were performed with four biological replications and three technical replications.

### Microscopy analysis of chloroplast

Transmission electron microscopy was ultilized to observe the ultrastructure of chloroplasts. Samples were prepared following Cao’s method [39]. To further observe the chloroplasts, protoplasts were isolated according to the procedure of Yoo et al. [40]. The protoplasts were recorded with inverted microscope (Olympus 1 × 71, Japan).

### BSR-seq analysis

Bulked segregant analysis in combination with RNA-seq (BSR-seq) [18] were employed to identify the albino trait. For BSR-seq, two pools, the albino pool (A-pool, mutant pool) and the normal pool (N-pool, wild pool), were constructed by mixing an equal amount of tissues from 50 individuals of albino leaves and 50 individuals of normal leaves in the BC_1_ population, respectively. Total RNA was extracted from the two pools to accomplish the RNA sequencing using RNAiso plus kit method (Takara, Japan).

Pair-end (125 bp) libraries with insert sizes of approximate 350 bp were prepared for sequencing on the Illumina Hiseq™ 2500 platform. Approximate 5 GB clean data were generated by RNA-seq for each pool (NCBI Submission Archive, PRJNA580294). The alignments of paired-end reads were processed by the Hisat2 program [41] against reference genome 02–12 [42], and SNP callings were performed using SAMtools [43]. SNP-index and Δ (SNP-index) were computed to identify the candidate regions for the albino trait. The key parameter of the Δ (SNP-index) was calculated by subtracting the SNP-index of the N-pool from the SNP-index of the A-pool. Confidence intervals (CIs) of 95 and 99% were computed for Δ (SNP-index) as described in a previous study [22]. The differentially expressed genes were analysed by FPKM (fragments per kilobase of transcript per million read pairs) values using the StringTie program [44].

### Molecular marker development and genetic mapping

Several types of molecular markers were used in the *AK* mapping (Table S2), including CAPS, Presence/absence, and SNP. Sequence specific primers for these markers were designed by using Primer 3.0 (http://primer3.ut.ee/) according to reference genome 02–12 [42]. A total of 93 randomly selected progenies in the BC_1_ population were used for preliminary mapping. The *AK* gene was fine-mapped with 603 progenies of the BC_1_ population. Genotyping data of the BC_1_ individuals were utilized for linkage analysis by JoinMap4 program [45].

### Candidate gene prediction

The differentially expressed genes were identified by StingTie [44] to get more information for the candidate gene selection. The genomic sequences in the target region were extracted from reference genome 02–12, and they were further predicted the Open Reading Frames (ORFs) by FGENESH (http://www.softberry.com/) and GENESCAN (http://genes.mit.edu/). The function annotations of these genes were retrieved from Blast2Go software (https://www.blast2go.com/) and TAIR website (https://www.arabidopsis.org/).

### Gene expression analysis

Total RNA was isolated from fresh tissues with RNAiso plus reagent (Takara, Japan). The quality of RNA was measured by NanoDrop 2000 (ThermoFisher Scientific, USA). Furthermore, the value of 260/280 > 1.8 was required for RNA samples in this study. The first-strand cDNA was synthesized by 2 μg RNA using TransScript One-Step gDNA Removal and cDNA Synthesis SuperMix kit (TransScript, China). The synthesized cDNAs were diluted with ddH_2_O for RT-PCR. The quantitative RT-PCR was performed in QuantStudio 5 Real-Time PCR Systems (ThermoFisher Scientific, USA). The reaction volume was 10 μL containing 5 μL SYBR qPCR Master Mix (Vazyme, China), 0.3 μL forward primer and reverse primer with 10 μM concentration, 1 μL cDNA template, and 3.4 μL ddH_2_O. PCR amplification was processed by two-step cycling method of 95 °C for 30s, and followed by 40 cycles of 95 °C for 5 s, and 60 °C for 20s. Melting curve was utilized to verify the specificity of primers. The expression levels of the candidate gene *Bol015404* (qP450-F: GGGAAACATCCACAAGCACA; qP450-R: TCTTTGGCCAGCCTTCAAAT) were detected with three biological replicates and three technical replications in several tissues including root, stem, inner leaves and outer leaves at 10 °C. The cabbage *β*-*actin* gene (AF044573) was used as the internal reference gene [46]. The relative expression levels were calculated with the formula 2^-△△Cq^ [17]. Student’s *t* test was used to estimate significant differences among different samples.

## Supplementary information

**Additional file 1: Table S1**. The phenotypes of albino and anthocyanin traits in the BC1 population. **Table S2.** Molecular markers for mapping of *AK* in C03. **Figure S1.** The phenotypes of albino and normal individuals in the BC_1_ population. **Figure S2** The phenotypes of albino, slight albino and normal individuals in the BC_1_F_2_ population. **Figure S3.** The alignment of coding sequences of *Bol015404* alleles in WK02 (WK), RK01 (RK) and green cabbage (GC). Text S1 Genomic sequences of *Bol015404* for RK01 (RK), WK01 (WK) and green cabbage(GC).

## Data Availability

The datasets generated during the current study are available in the NCBI (PRJNA580294).

## Abbreviations

CTIC: Cool-Temperature-Induced Chlorosis
BSR-seq: BSA + RNA-seq
SNP: Single Nucleotide Polymorphisim
CAPS: C1eaved Amplified PolymorphicSequences
LD: Long Day
SD: Short Day
CIs: Confidence Intervals
