# Supplementary material for: Fine mapping of a candidate gene for cool-temperature-induced albinism in ornamental kale
Source: BMC Plant Biol. 2020 Oct 7;20:460. doi: 10.1186/s12870-020-02657-0 (PMC7541286; doi:10.1186/s12870-020-02657-0)
Supplement: Supplementary file 1 — Additional file 1: Table S1. The phenotypes of albino and anthocyanin traits in the BC1 population. Table S2. Molecular markers for mapping of AK in C03. Figure S1. The phenotypes of albino and normal individuals in the BC1 population. Figure S2 The phenotypes of albino, slight albino and normal individuals in the BC1F2 population. Figure S3. The alignment of coding sequences of Bol015404 alleles in WK02 (WK), RK01 (RK) and green cabbage (GC). Text S1 Genomic sequences of Bol015404 for RK01 (RK), WK01 (WK) and green cabbage(GC). [file 12870_2020_2657_MOESM1_ESM.docx]

Supplementary information for:

**Fine mapping of a candidate gene for cool-temperature-induced** **albinism in ornamental kale**

Chenghuan Yan^1,2^, Liying Peng^1^, Lei Zhang^1^, Zhengming Qiu^2^*

^1^Key Laboratory of Horticultural Plant Biology, Ministry of Education, College of Horticulture and Forestry Sciences, Huazhong Agricultural University, Wuhan 430070, People’s Republic of China

^2^Hubei Key Laboratory of vegetable Germplasm Enhancement and Genetic Improvement, Institute of Economic Crops, Hubei Academy of Agricultural Sciences, Wuhan 430064, People’s Republic of China

***Author for correspondence:**

**Zhengming Qiu**: qiusunmoon@163.com

**Tables and Figures Legends**

**Table S1 The phenotypes of albino and anthocyanin traits in the BC1 population**

| No. | Plants | Albinism | Anthocyanin | Two_Traits_Combined |
| --- | --- | --- | --- | --- |
| P1 | Red Kamome | A | Yes | Red |
| P2 | HGDH | N | No | Green |
| F1 | P1XP2 | SN | Yes | Red |
| 1 | BC1-1 | A | Yes | Red |
| 2 | BC1-2 | A | Yes | Red |
| 3 | BC1-3 | A | No | White |
| 4 | BC1-4 | N | No | Green |
| 5 | BC1-5 | A | Yes | Red |
| 6 | BC1-6 | A | No | White |
| 7 | BC1-7 | N | No | Green |
| 8 | BC1-8 | N | No | Green |
| 9 | BC1-9 | A | No | White |
| 10 | BC1-10 | A | No | White |
| 11 | BC1-11 | N | No | Green |
| 12 | BC1-12 | A | Yes | Red |
| 13 | BC1-13 | A | No | White |
| 14 | BC1-14 | N | No | Green |
| 15 | BC1-15 | N | No | Green |
| 16 | BC1-16 | A | No | White |
| 17 | BC1-17 | N | Yes | Purple |
| 18 | BC1-18 | A | Yes | Red |
| 19 | BC1-19 | A | Yes | Red |
| 20 | BC1-20 | N | Yes | Purple |
| 21 | BC1-21 | A | Yes | Red |
| 22 | BC1-22 | A | Yes | Red |
| 23 | BC1-23 | N | Yes | Purple |
| 24 | BC1-24 | N | No | Green |
| 25 | BC1-25 | A | Yes | Red |
| 26 | BC1-26 | N | No | Green |
| 27 | BC1-27 | A | Yes | Red |
| 28 | BC1-28 | N | Yes | Purple |
| 29 | BC1-29 | N | No | Green |
| 30 | BC1-30 | N | Yes | Purple |
| 31 | BC1-31 | A | No | White |
| 32 | BC1-32 | N | No | Green |
| 33 | BC1-33 | N | No | Green |
| 34 | BC1-34 | N | No | Green |
| 35 | BC1-35 | N | Yes | Purple |
| 36 | BC1-36 | A | No | White |
| 37 | BC1-37 | N | No | Green |
| 38 | BC1-38 | A | No | White |
| 39 | BC1-39 | A | No | White |
| 40 | BC1-40 | N | Yes | Purple |
| 41 | BC1-41 | N | Yes | Purple |
| 42 | BC1-42 | A | No | White |
| 43 | BC1-43 | A | No | White |
| 44 | BC1-44 | N | No | Green |
| 45 | BC1-45 | N | Yes | Purple |
| 46 | BC1-46 | N | No | Green |
| 47 | BC1-47 | A | No | White |
| 48 | BC1-48 | A | Yes | Red |
| 49 | BC1-49 | N | No | Green |
| 50 | BC1-50 | N | Yes | Purple |
| 51 | BC1-51 | N | No | Green |
| 52 | BC1-52 | A | No | White |
| 53 | BC1-53 | N | No | Green |
| 54 | BC1-54 | N | No | Green |
| 55 | BC1-55 | A | Yes | Red |
| 56 | BC1-56 | N | No | Green |
| 57 | BC1-57 | A | No | White |
| 58 | BC1-58 | A | Yes | Red |
| 59 | BC1-59 | N | No | Green |
| 60 | BC1-60 | N | Yes | Purple |
| 61 | BC1-61 | N | Yes | Purple |
| 62 | BC1-62 | N | No | Green |
| 63 | BC1-63 | N | No | Green |
| 64 | BC1-64 | A | Yes | Red |
| 65 | BC1-65 | N | No | Green |
| 66 | BC1-66 | A | Yes | Red |
| 67 | BC1-67 | A | Yes | Red |
| 68 | BC1-68 | N | No | Green |
| 69 | BC1-69 | N | No | Green |
| 70 | BC1-70 | N | No | Green |
| 71 | BC1-71 | N | Yes | Purple |
| 72 | BC1-72 | A | Yes | Red |
| 73 | BC1-73 | N | Yes | Purple |
| 74 | BC1-74 | N | No | Green |
| 75 | BC1-75 | A | Yes | Red |
| 76 | BC1-76 | N | Yes | Purple |
| 77 | BC1-77 | N | Yes | Purple |
| 78 | BC1-78 | A | No | White |
| 79 | BC1-79 | A | Yes | Red |
| 80 | BC1-80 | N | Yes | Purple |
| 81 | BC1-81 | N | No | Green |
| 82 | BC1-82 | A | No | White |
| 83 | BC1-83 | A | Yes | Red |
| 84 | BC1-84 | N | No | Green |
| 85 | BC1-85 | N | Yes | Purple |
| 86 | BC1-86 | A | No | White |
| 87 | BC1-87 | A | Yes | Red |
| 88 | BC1-88 | A | No | White |
| 89 | BC1-89 | N | Yes | Purple |
| 90 | BC1-90 | N | No | Green |
| 91 | BC1-91 | A | No | White |
| 92 | BC1-92 | A | Yes | Red |
| 93 | BC1-93 | N | Yes | Purple |
| 94 | BC1-94 | N | No | Green |
| 95 | BC1-95 | N | Yes | Purple |
| 96 | BC1-96 | N | No | Green |
| 97 | BC1-97 | N | Yes | Purple |
| 98 | BC1-98 | A | Yes | Red |
| 99 | BC1-99 | A | No | White |
| 100 | BC1-100 | N | Yes | Purple |
| 101 | BC1-101 | A | No | White |
| 102 | BC1-102 | N | Yes | Purple |
| 103 | BC1-103 | A | No | White |
| 104 | BC1-104 | A | No | White |
| 105 | BC1-105 | A | No | White |
| 106 | BC1-106 | A | No | White |
| 107 | BC1-107 | A | Yes | Red |
| 108 | BC1-108 | A | Yes | Red |
| 109 | BC1-109 | A | No | White |
| 110 | BC1-110 | N | No | Green |
| 111 | BC1-111 | N | Yes | Purple |
| 112 | BC1-112 | A | No | White |
| 113 | BC1-113 | N | No | Green |
| 114 | BC1-114 | N | No | Green |
| 115 | BC1-115 | A | Yes | Red |
| 116 | BC1-116 | A | Yes | Red |
| 117 | BC1-117 | N | Yes | Purple |
| 118 | BC1-118 | N | No | Green |
| 119 | BC1-119 | N | Yes | Purple |
| 120 | BC1-120 | A | Yes | Red |
| 121 | BC1-121 | A | Yes | Red |
| 122 | BC1-122 | N | No | Green |
| 123 | BC1-123 | N | Yes | Purple |
| 124 | BC1-124 | N | Yes | Purple |
| 125 | BC1-125 | A | Yes | Red |
| 126 | BC1-126 | A | Yes | Red |
| 127 | BC1-127 | A | No | White |
| 128 | BC1-128 | A | No | White |
| 129 | BC1-129 | N | No | Green |
| 130 | BC1-130 | N | No | Green |
| 131 | BC1-131 | A | No | White |
| 132 | BC1-132 | A | No | White |
| 133 | BC1-133 | A | No | White |
| 134 | BC1-134 | A | Yes | Red |
| 135 | BC1-135 | N | No | Green |
| 136 | BC1-136 | A | Yes | Red |
| 137 | BC1-137 | N | No | Green |
| 138 | BC1-138 | A | Yes | Red |
| 139 | BC1-139 | A | No | White |
| 140 | BC1-140 | N | No | Green |
| 141 | BC1-141 | A | Yes | Red |
| 142 | BC1-142 | A | No | White |
| 143 | BC1-143 | A | Yes | Red |
| 144 | BC1-144 | A | Yes | Red |
| 145 | BC1-145 | A | Yes | Red |
| 146 | BC1-146 | N | Yes | Purple |
| 147 | BC1-147 | N | Yes | Purple |
| 148 | BC1-148 | A | Yes | Red |
| 149 | BC1-149 | N | Yes | Purple |
| 150 | BC1-150 | A | Yes | Red |
| 151 | BC1-151 | A | Yes | Red |
| 152 | BC1-152 | N | No | Green |
| 153 | BC1-153 | N | No | Green |
| 154 | BC1-154 | A | No | White |
| 155 | BC1-155 | N | Yes | Purple |
| 156 | BC1-156 | N | No | Green |
| 157 | BC1-157 | N | Yes | Purple |
| 158 | BC1-158 | A | Yes | Red |
| 159 | BC1-159 | N | No | Green |
| 160 | BC1-160 | N | No | Green |
| 161 | BC1-161 | A | No | White |
| 162 | BC1-162 | A | No | White |
| 163 | BC1-163 | N | No | Green |
| 164 | BC1-164 | N | Yes | Purple |
| 165 | BC1-165 | A | No | White |
| 166 | BC1-166 | A | No | White |
| 167 | BC1-167 | N | No | Green |
| 168 | BC1-168 | A | No | White |
| 169 | BC1-169 | N | No | Green |
| 170 | BC1-170 | A | No | White |
| 171 | BC1-171 | N | Yes | Purple |
| 172 | BC1-172 | N | No | Green |
| 173 | BC1-173 | N | No | Green |
| 174 | BC1-174 | A | Yes | Red |
| 175 | BC1-175 | N | No | Green |
| 176 | BC1-176 | A | Yes | Red |
| 177 | BC1-177 | N | No | Green |
| 178 | BC1-178 | A | No | White |
| 179 | BC1-179 | N | Yes | Purple |
| 180 | BC1-180 | A | No | White |
| 181 | BC1-181 | N | Yes | Purple |
| 182 | BC1-182 | A | Yes | Red |
| 183 | BC1-183 | N | Yes | Purple |
| 184 | BC1-184 | N | No | Green |
| 185 | BC1-185 | N | Yes | Purple |
| 186 | BC1-186 | N | Yes | Purple |
| 187 | BC1-187 | A | No | White |
| 188 | BC1-188 | N | Yes | Purple |
| 189 | BC1-189 | A | Yes | Red |
| 190 | BC1-190 | N | Yes | Purple |
| 191 | BC1-191 | N | No | Green |
| 192 | BC1-192 | A | Yes | Red |
| 193 | BC1-193 | A | Yes | Red |
| 194 | BC1-194 | N | No | Green |
| 195 | BC1-195 | A | No | White |
| 196 | BC1-196 | A | No | White |
| 197 | BC1-197 | N | No | Green |
| 198 | BC1-198 | A | No | White |
| 199 | BC1-199 | N | Yes | Purple |
| 200 | BC1-200 | N | No | Green |
| 201 | BC1-201 | A | Yes | Red |
| 202 | BC1-202 | A | Yes | Red |
| 203 | BC1-203 | A | No | White |
| 204 | BC1-204 | A | No | White |
| 205 | BC1-205 | A | No | White |
| 206 | BC1-206 | A | Yes | Red |
| 207 | BC1-207 | A | No | White |
| 208 | BC1-208 | A | No | White |
| 209 | BC1-209 | N | No | Green |
| 210 | BC1-210 | N | No | Green |
| 211 | BC1-211 | A | No | White |
| 212 | BC1-212 | N | No | Green |
| 213 | BC1-213 | N | No | Green |
| 214 | BC1-214 | N | Yes | Purple |
| 215 | BC1-215 | A | No | White |
| 216 | BC1-216 | A | No | White |
| 217 | BC1-217 | N | Yes | Purple |
| 218 | BC1-218 | A | No | White |
| 219 | BC1-219 | N | Yes | Purple |
| 220 | BC1-220 | N | No | Green |
| 221 | BC1-221 | A | Yes | Red |
| 222 | BC1-222 | A | Yes | Red |
| 223 | BC1-223 | N | Yes | Purple |
| 224 | BC1-224 | N | Yes | Purple |
| 225 | BC1-225 | N | Yes | Purple |
| 226 | BC1-226 | N | Yes | Purple |
| 227 | BC1-227 | A | No | White |
| 228 | BC1-228 | N | No | Green |
| 229 | BC1-229 | A | Yes | Red |
| 230 | BC1-230 | A | No | White |
| 231 | BC1-231 | N | No | Green |
| 232 | BC1-232 | A | No | White |
| 233 | BC1-233 | N | Yes | Purple |
| 234 | BC1-234 | N | Yes | Purple |
| 235 | BC1-235 | A | No | White |
| 236 | BC1-236 | A | No | White |
| 237 | BC1-237 | N | No | Green |
| 238 | BC1-238 | N | No | Green |
| 239 | BC1-239 | N | Yes | Purple |
| 240 | BC1-240 | A | Yes | Red |
| 241 | BC1-241 | A | Yes | Red |
| 242 | BC1-242 | N | No | Green |
| 243 | BC1-243 | N | Yes | Purple |
| 244 | BC1-244 | A | No | White |
| 245 | BC1-245 | N | Yes | Purple |
| 246 | BC1-246 | A | Yes | Red |
| 247 | BC1-247 | N | No | Green |
| 248 | BC1-248 | N | No | Green |
| 249 | BC1-249 | N | Yes | Purple |
| 250 | BC1-250 | A | No | White |
| 251 | BC1-251 | A | Yes | Red |
| 252 | BC1-252 | N | Yes | Purple |
| 253 | BC1-253 | N | No | Green |
| 254 | BC1-254 | A | Yes | Red |
| 255 | BC1-255 | N | No | Green |
| 256 | BC1-256 | A | No | White |
| 257 | BC1-257 | A | No | White |
| 258 | BC1-258 | N | Yes | Purple |
| 259 | BC1-259 | A | Yes | Red |
| 260 | BC1-260 | A | No | White |
| 261 | BC1-261 | N | No | Green |
| 262 | BC1-262 | N | Yes | Purple |
| 263 | BC1-263 | A | Yes | Red |
| 264 | BC1-264 | A | Yes | Red |
| 265 | BC1-265 | N | No | Green |
| 266 | BC1-266 | N | No | Green |
| 267 | BC1-267 | A | Yes | Red |
| 268 | BC1-268 | A | Yes | Red |
| 269 | BC1-269 | A | No | White |
| 270 | BC1-270 | N | No | Green |
| 271 | BC1-271 | N | No | Green |
| 272 | BC1-272 | A | No | White |
| 273 | BC1-273 | N | Yes | Purple |
| 274 | BC1-274 | N | No | Green |
| 275 | BC1-275 | A | Yes | Red |
| 276 | BC1-276 | A | Yes | Red |
| 277 | BC1-277 | A | No | White |
| 278 | BC1-278 | N | Yes | Purple |
| 279 | BC1-279 | A | No | White |
| 280 | BC1-280 | N | No | Green |
| 281 | BC1-281 | A | No | White |
| 282 | BC1-282 | N | Yes | Purple |
| 283 | BC1-283 | A | Yes | Red |
| 284 | BC1-284 | N | Yes | Purple |
| 285 | BC1-285 | A | No | White |
| 286 | BC1-286 | N | No | Green |
| 287 | BC1-287 | A | No | White |
| 288 | BC1-288 | A | No | White |
| 289 | BC1-289 | N | No | Green |
| 290 | BC1-290 | A | Yes | Red |
| 291 | BC1-291 | N | No | Green |
| 292 | BC1-292 | A | No | White |
| 293 | BC1-293 | N | No | Green |
| 294 | BC1-294 | A | Yes | Red |
| 295 | BC1-295 | N | No | Green |
| 296 | BC1-296 | N | No | Green |
| 297 | BC1-297 | A | No | White |
| 298 | BC1-298 | A | No | White |
| 299 | BC1-299 | N | No | Green |
| 300 | BC1-300 | N | Yes | Purple |
| 301 | BC1-301 | A | Yes | Red |
| 302 | BC1-302 | A | Yes | Red |
| 303 | BC1-303 | N | Yes | Purple |
| 304 | BC1-304 | N | No | Green |
| 305 | BC1-305 | A | No | White |
| 306 | BC1-306 | N | Yes | Purple |
| 307 | BC1-307 | A | Yes | Red |
| 308 | BC1-308 | A | Yes | Red |
| 309 | BC1-309 | N | No | Green |
| 310 | BC1-310 | N | Yes | Purple |
| 311 | BC1-311 | A | Yes | Red |
| 312 | BC1-312 | N | No | Green |
| 313 | BC1-313 | N | No | Green |
| 314 | BC1-314 | N | Yes | Purple |
| 315 | BC1-315 | N | No | Green |
| 316 | BC1-316 | N | Yes | Purple |
| 317 | BC1-317 | A | Yes | Red |
| 318 | BC1-318 | A | Yes | Red |
| 319 | BC1-319 | N | No | Green |
| 320 | BC1-320 | A | Yes | Red |
| 321 | BC1-321 | A | Yes | Red |
| 322 | BC1-322 | N | No | Green |
| 323 | BC1-323 | A | Yes | Red |
| 324 | BC1-324 | A | Yes | Red |
| 325 | BC1-325 | A | No | White |
| 326 | BC1-326 | N | No | Green |
| 327 | BC1-327 | A | No | White |
| 328 | BC1-328 | A | No | White |
| 329 | BC1-329 | N | No | Green |
| 330 | BC1-330 | N | Yes | Purple |
| 331 | BC1-331 | N | No | Green |
| 332 | BC1-332 | N | Yes | Purple |
| 333 | BC1-333 | A | Yes | Red |
| 334 | BC1-334 | N | No | Green |
| 335 | BC1-335 | A | Yes | Red |
| 336 | BC1-336 | N | Yes | Purple |
| 337 | BC1-337 | N | Yes | Purple |
| 338 | BC1-338 | A | No | White |
| 339 | BC1-339 | A | Yes | Red |
| 340 | BC1-340 | N | No | Green |
| 341 | BC1-341 | A | No | White |
| 342 | BC1-342 | A | Yes | Red |
| 343 | BC1-343 | N | No | Green |
| 344 | BC1-344 | N | Yes | Purple |
| 345 | BC1-345 | N | Yes | Purple |
| 346 | BC1-346 | A | No | White |
| 347 | BC1-347 | A | No | White |
| 348 | BC1-348 | A | No | White |
| 349 | BC1-349 | A | Yes | Red |
| 350 | BC1-350 | N | Yes | Purple |
| 351 | BC1-351 | N | Yes | Purple |
| 352 | BC1-352 | N | No | Green |
| 353 | BC1-353 | A | No | White |
| 354 | BC1-354 | A | No | White |
| 355 | BC1-355 | N | Yes | Purple |
| 356 | BC1-356 | N | No | Green |
| 357 | BC1-357 | N | No | Green |
| 358 | BC1-358 | A | Yes | Red |
| 359 | BC1-359 | A | Yes | Red |
| 360 | BC1-360 | N | Yes | Purple |
| 361 | BC1-361 | N | Yes | Purple |
| 362 | BC1-362 | A | Yes | Red |
| 363 | BC1-363 | N | Yes | Purple |
| 364 | BC1-364 | A | No | White |
| 365 | BC1-365 | N | No | Green |
| 366 | BC1-366 | A | Yes | Red |
| 367 | BC1-367 | A | Yes | Red |
| 368 | BC1-368 | A | No | White |
| 369 | BC1-369 | A | Yes | Red |
| 370 | BC1-370 | A | No | White |
| 371 | BC1-371 | A | No | White |
| 372 | BC1-372 | N | No | Green |
| 373 | BC1-373 | N | No | Green |
| 374 | BC1-374 | N | Yes | Purple |
| 375 | BC1-375 | A | Yes | Red |
| 376 | BC1-376 | A | Yes | Red |
| 377 | BC1-377 | N | Yes | Purple |
| 378 | BC1-378 | N | No | Green |
| 379 | BC1-379 | A | Yes | Red |
| 380 | BC1-380 | A | Yes | Red |
| 381 | BC1-381 | A | No | White |
| 382 | BC1-382 | A | Yes | Red |
| 383 | BC1-383 | N | Yes | Purple |
| 384 | BC1-384 | N | No | Green |
| 385 | BC1-385 | A | No | White |
| 386 | BC1-386 | N | No | Green |
| 387 | BC1-387 | A | Yes | Red |
| 388 | BC1-388 | A | Yes | Red |
| 389 | BC1-389 | A | No | White |
| 390 | BC1-390 | N | Yes | Purple |
| 391 | BC1-391 | A | Yes | Red |
| 392 | BC1-392 | A | Yes | Red |
| 393 | BC1-393 | N | No | Green |
| 394 | BC1-394 | A | No | White |
| 395 | BC1-395 | N | No | Green |
| 396 | BC1-396 | N | No | Green |
| 397 | BC1-397 | N | No | Green |
| 398 | BC1-398 | N | No | Green |
| 399 | BC1-399 | A | Yes | Red |
| 400 | BC1-400 | N | Yes | Purple |
| 401 | BC1-401 | A | No | White |
| 402 | BC1-402 | A | Yes | Red |
| 403 | BC1-403 | A | No | White |
| 404 | BC1-404 | A | Yes | Red |
| 405 | BC1-405 | N | Yes | Purple |
| 406 | BC1-406 | N | No | Green |
| 407 | BC1-407 | A | Yes | Red |
| 408 | BC1-408 | A | Yes | Red |
| 409 | BC1-409 | A | Yes | Red |
| 410 | BC1-410 | A | Yes | Red |
| 411 | BC1-411 | N | No | Green |
| 412 | BC1-412 | A | Yes | Red |
| 413 | BC1-413 | N | No | Green |
| 414 | BC1-414 | A | Yes | Red |
| 415 | BC1-415 | A | No | White |
| 416 | BC1-416 | N | No | Green |
| 417 | BC1-417 | A | No | White |
| 418 | BC1-418 | A | Yes | Red |
| 419 | BC1-419 | A | No | White |
| 420 | BC1-420 | N | No | Green |
| 421 | BC1-421 | A | Yes | Red |
| 422 | BC1-422 | N | Yes | Purple |
| 423 | BC1-423 | A | No | White |
| 424 | BC1-424 | N | No | Green |
| 425 | BC1-425 | N | Yes | Purple |
| 426 | BC1-426 | N | No | Green |
| 427 | BC1-427 | A | No | White |
| 428 | BC1-428 | A | No | White |
| 429 | BC1-429 | A | Yes | Red |
| 430 | BC1-430 | N | No | Green |
| 431 | BC1-431 | N | Yes | Purple |
| 432 | BC1-432 | A | No | White |
| 433 | BC1-433 | N | Yes | Purple |
| 434 | BC1-434 | N | Yes | Purple |
| 435 | BC1-435 | A | No | White |
| 436 | BC1-436 | N | Yes | Purple |
| 437 | BC1-437 | A | Yes | Red |
| 438 | BC1-438 | N | No | Green |
| 439 | BC1-439 | A | Yes | Red |
| 440 | BC1-440 | A | Yes | Red |
| 441 | BC1-441 | N | No | Green |
| 442 | BC1-442 | N | No | Green |
| 443 | BC1-443 | N | No | Green |
| 444 | BC1-444 | A | No | White |
| 445 | BC1-445 | N | Yes | Purple |
| 446 | BC1-446 | N | No | Green |
| 447 | BC1-447 | A | Yes | Red |
| 448 | BC1-448 | A | Yes | Red |
| 449 | BC1-449 | A | Yes | Red |
| 450 | BC1-450 | A | No | White |
| 451 | BC1-451 | N | Yes | Purple |
| 452 | BC1-452 | N | No | Green |
| 453 | BC1-453 | N | Yes | Purple |
| 454 | BC1-454 | A | Yes | Red |
| 455 | BC1-455 | N | Yes | Purple |
| 456 | BC1-456 | N | No | Green |
| 457 | BC1-457 | N | Yes | Purple |
| 458 | BC1-458 | N | No | Green |
| 459 | BC1-459 | A | No | White |
| 460 | BC1-460 | A | Yes | Red |
| 461 | BC1-461 | N | Yes | Purple |
| 462 | BC1-462 | A | Yes | Red |
| 463 | BC1-463 | A | Yes | Red |
| 464 | BC1-464 | N | Yes | Purple |
| 465 | BC1-465 | N | Yes | Purple |
| 466 | BC1-466 | A | No | White |
| 467 | BC1-467 | N | Yes | Purple |
| 468 | BC1-468 | A | No | White |
| 469 | BC1-469 | N | Yes | Purple |
| 470 | BC1-470 | A | Yes | Red |
| 471 | BC1-471 | A | No | White |
| 472 | BC1-472 | A | Yes | Red |
| 473 | BC1-473 | N | No | Green |
| 474 | BC1-474 | N | Yes | Purple |
| 475 | BC1-475 | A | Yes | Red |
| 476 | BC1-476 | A | Yes | Red |
| 477 | BC1-477 | A | No | White |
| 478 | BC1-478 | A | Yes | Red |
| 479 | BC1-479 | N | Yes | Purple |
| 480 | BC1-480 | N | Yes | Purple |
| 481 | BC1-481 | A | No | White |
| 482 | BC1-482 | N | No | Green |
| 483 | BC1-483 | A | No | White |
| 484 | BC1-484 | N | No | Green |
| 485 | BC1-485 | N | Yes | Purple |
| 486 | BC1-486 | A | No | White |
| 487 | BC1-487 | N | No | Green |
| 488 | BC1-488 | N | No | Green |
| 489 | BC1-489 | A | Yes | Red |
| 490 | BC1-490 | N | No | Green |
| 491 | BC1-491 | A | Yes | Red |
| 492 | BC1-492 | A | No | White |
| 493 | BC1-493 | A | No | White |
| 494 | BC1-494 | N | Yes | Purple |
| 495 | BC1-495 | A | No | White |
| 496 | BC1-496 | N | No | Green |
| 497 | BC1-497 | A | No | White |
| 498 | BC1-498 | N | Yes | Purple |
| 499 | BC1-499 | A | No | White |
| 500 | BC1-500 | A | No | White |
| 501 | BC1-501 | A | No | White |
| 502 | BC1-502 | N | Yes | Purple |
| 503 | BC1-503 | A | Yes | Red |
| 504 | BC1-504 | N | Yes | Purple |
| 505 | BC1-505 | A | Yes | Red |
| 506 | BC1-506 | A | Yes | Red |
| 507 | BC1-507 | N | Yes | Purple |
| 508 | BC1-508 | N | No | Green |
| 509 | BC1-509 | N | No | Green |
| 510 | BC1-510 | N | Yes | Purple |
| 511 | BC1-511 | N | No | Green |
| 512 | BC1-512 | N | No | Green |
| 513 | BC1-513 | N | No | Green |
| 514 | BC1-514 | N | No | Green |
| 515 | BC1-515 | N | No | Green |
| 516 | BC1-516 | N | Yes | Purple |
| 517 | BC1-517 | A | Yes | Red |
| 518 | BC1-518 | N | Yes | Purple |
| 519 | BC1-519 | A | Yes | Red |
| 520 | BC1-520 | A | Yes | Red |
| 521 | BC1-521 | N | No | Green |
| 522 | BC1-522 | A | No | White |
| 523 | BC1-523 | A | No | White |
| 524 | BC1-524 | N | No | Green |
| 525 | BC1-525 | N | Yes | Purple |
| 526 | BC1-526 | N | Yes | Purple |
| 527 | BC1-527 | A | No | White |
| 528 | BC1-528 | N | No | Green |
| 529 | BC1-529 | N | No | Green |
| 530 | BC1-530 | N | No | Green |
| 531 | BC1-531 | N | No | Green |
| 532 | BC1-532 | A | No | White |
| 533 | BC1-533 | A | Yes | Red |
| 534 | BC1-534 | A | No | White |
| 535 | BC1-535 | N | Yes | Purple |
| 536 | BC1-536 | A | Yes | Red |
| 537 | BC1-537 | A | Yes | Red |
| 538 | BC1-538 | A | Yes | Red |
| 539 | BC1-539 | A | Yes | Red |
| 540 | BC1-540 | N | Yes | Purple |
| 541 | BC1-541 | A | No | White |
| 542 | BC1-542 | A | No | White |
| 543 | BC1-543 | A | Yes | Red |
| 544 | BC1-544 | A | Yes | Red |
| 545 | BC1-545 | N | No | Green |
| 546 | BC1-546 | N | No | Green |
| 547 | BC1-547 | A | Yes | Red |
| 548 | BC1-548 | N | No | Green |
| 549 | BC1-549 | A | Yes | Red |
| 550 | BC1-550 | A | Yes | Red |
| 551 | BC1-551 | A | Yes | Red |
| 552 | BC1-552 | N | Yes | Purple |
| 553 | BC1-553 | A | No | White |
| 554 | BC1-554 | N | Yes | Purple |
| 555 | BC1-555 | A | Yes | Red |
| 556 | BC1-556 | A | No | White |
| 557 | BC1-557 | N | No | Green |
| 558 | BC1-558 | N | Yes | Purple |
| 559 | BC1-559 | N | Yes | Purple |
| 560 | BC1-560 | N | Yes | Purple |
| 561 | BC1-561 | N | Yes | Purple |
| 562 | BC1-562 | N | Yes | Purple |
| 563 | BC1-563 | A | No | White |
| 564 | BC1-564 | N | Yes | Purple |
| 565 | BC1-565 | A | No | White |
| 566 | BC1-566 | N | Yes | Purple |
| 567 | BC1-567 | A | Yes | Red |
| 568 | BC1-568 | N | No | Green |
| 569 | BC1-569 | N | Yes | Purple |
| 570 | BC1-570 | N | No | Green |
| 571 | BC1-571 | A | No | White |
| 572 | BC1-572 | N | No | Green |
| 573 | BC1-573 | A | Yes | Red |
| 574 | BC1-574 | A | Yes | Red |
| 575 | BC1-575 | A | No | White |
| 576 | BC1-576 | N | Yes | Purple |
| 577 | BC1-577 | A | No | White |
| 578 | BC1-578 | N | Yes | Purple |
| 579 | BC1-579 | A | No | White |
| 580 | BC1-580 | N | No | Green |
| 581 | BC1-581 | A | Yes | Red |
| 582 | BC1-582 | N | Yes | Purple |
| 583 | BC1-583 | A | Yes | Red |
| 584 | BC1-584 | A | No | White |
| 585 | BC1-585 | A | No | White |
| 586 | BC1-586 | N | Yes | Purple |
| 587 | BC1-587 | A | No | White |
| 588 | BC1-588 | N | No | Green |
| 589 | BC1-589 | A | Yes | Red |
| 590 | BC1-590 | A | No | White |
| 591 | BC1-591 | N | No | Green |
| 592 | BC1-592 | N | Yes | Purple |
| 593 | BC1-593 | N | Yes | Purple |
| 594 | BC1-594 | N | Yes | Purple |
| 595 | BC1-595 | A | No | White |
| 596 | BC1-596 | A | No | White |
| 597 | BC1-597 | A | No | White |
| 598 | BC1-598 | A | No | White |
| 599 | BC1-599 | A | No | White |
| 600 | BC1-600 | A | No | White |
| 601 | BC1-601 | N | No | Green |
| 602 | BC1-602 | A | No | White |
| 603 | BC1-603 | A | No | White |

Notes:

Albinism Trait A: Albino; N: Normal; SA: Slight albino.

Anthocyanin Trait Yes: Anthocyanin accumulation; No: No anthocyanin.

Two_Traits_Combined Purple: N+Yes; White: A+No; Red: A+Yes; Green: N+No.

**Table S2 Molecular markers for mapping of *AK* in C03**

| Name | Primers | Positions (C03, bp) | Types of molecular markers |
| --- | --- | --- | --- |
| BoY001-F | TCGCTCATGTTTCATAGGTTG | 300,692 | Presence/Absence |
| BoY001-R | GGGGTTTTCACAAAATCCAA |  |  |
| BoY002-F | GCAACAAGATCGCTGGATTC | 675,143 | CAPS(MboI) |
| BoY002-R | TCCTGATCGACTGATAACAACA |  |  |
| BoY003-F | GTGTTAGTGATCCAAATGTC | 1,229,918 | CAPS (BamHI) |
| BoY003-R | GCTTCTATTTTCCGGTCCAT |  |  |
| BoY006-F | AGCAACCGTTGAGCGTACTT | 3,617,374 | CAPS (HpaII) |
| BoY006-R | GAGCGCGTAGACTGAGACAA |  |  |
| BoY007-F | CATCTTCGGAAAGGCAACTC | 5,663,806 | CAPS (BamHI) |
| BoY007-R | GTTGTTGGCTCGGAGTTTGT |  |  |
| BoY008-F | TCGAACACACTGCATCATCA | 10,481,351 | CAPS (BamHI) |
| BoY008-R | AAGGAAGCTCTTTACCGCAAG |  |  |
| BoY009-F | TTGAGGAGATTGCTGGTGGA | 16,325,827 | CAPS (BsuRI) |
| BoY009-R | TCCTGCAAAATGACATGGCC |  |  |
| BoY010-F | AATCAGTCGTTTTCCTCTAT | 754,756 | SNP |
| BoY010-R | GTTCTTGAACAGGTTTCTCT |  |  |
| BoY011-F | GGGAATCCACAAACATCACA | 815,202 | CAPS (Bsh1236I) |
| BoY011-R | GCCATCTAAAGATTCGTTCTCC |  |  |
| BoY012-F | GAACGGAACACCGAGCTTTA | 928,595 | CAPS (Bsh1236I) |
| BoY012-R | TTCCTCCTTTCCAATCCAGA |  |  |
| BoY013-F | TCAGATGAGACCTTGACGTTT | 1,016,690 | CAPS (BamHI) |
| BoY013-R | CAGGAGCCAGACAGTGCTAA |  |  |
| BoY015-F | ATGCATTGTTCAAGGCACAACAAC | 805,407 | SNP |
| BoY015-R | CCGAAACAAAGGTCCATACC |  |  |


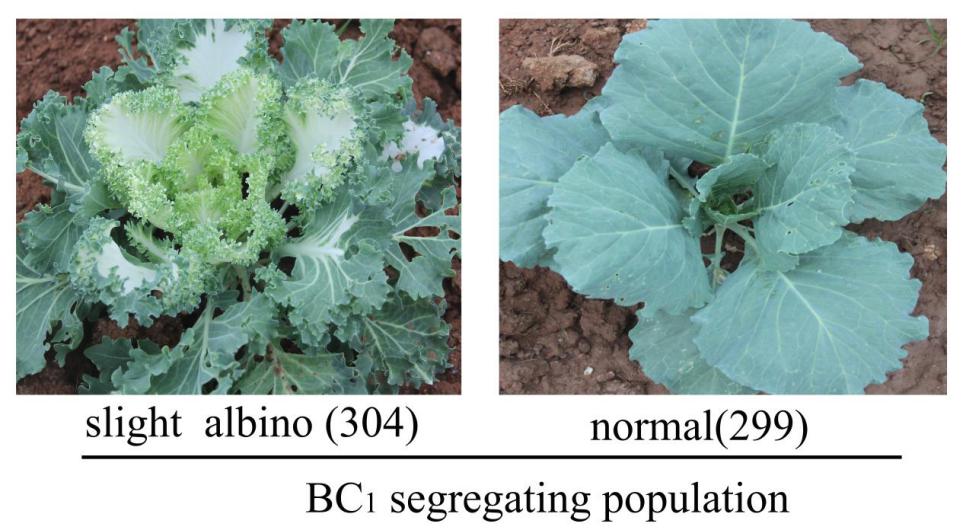


**Figure S1 The phenotypes of albino and normal individuals in the BC_1_ population.** The numbers in brackets represent the number of plants corresponding to the phenotypes.


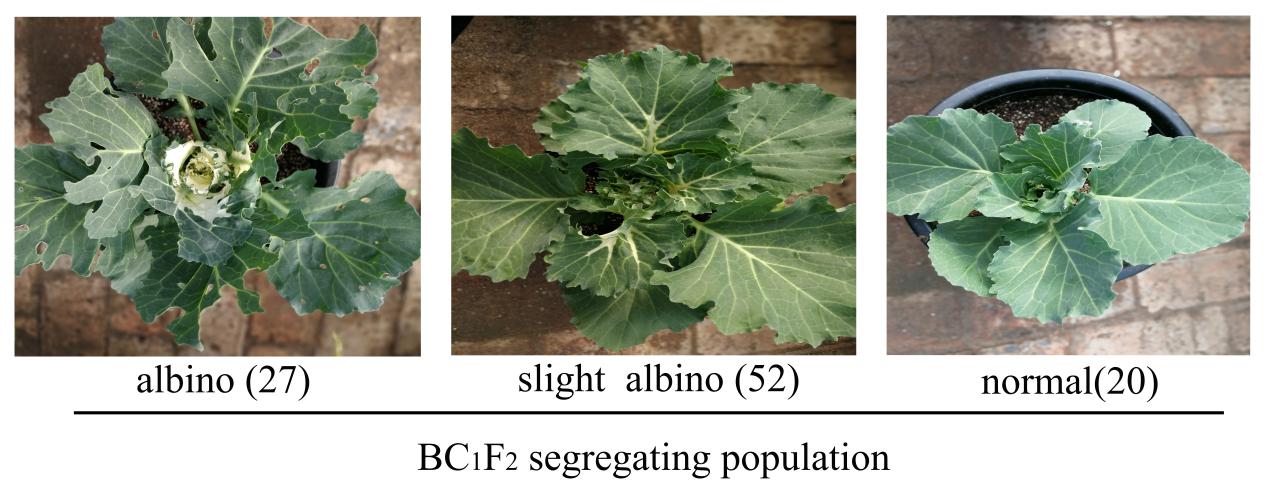


**Figure S2 The phenotypes of albino, slight albino and normal individuals in the BC_1_F_2_ population.** The numbers in brackets represent the number of plants corresponding to the phenotypes.


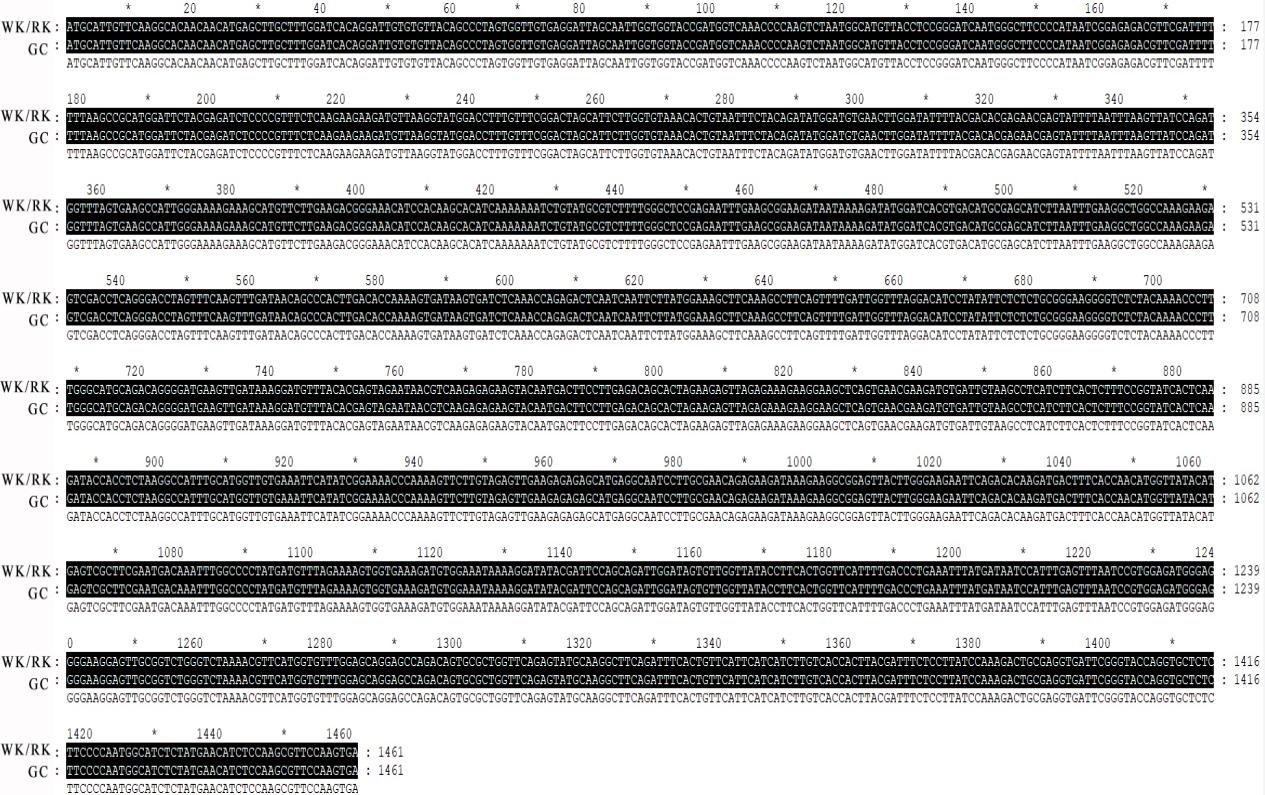


**Figure S3 The alignment of coding sequences of *Bol015404* alleles in WK02 (WK), RK01 (RK) and green cabbage (GC).**

**Text S1 Genomic sequences of *Bol015404* for RK01 (RK), WK01 (WK) and green cabbage(GC)**

> Bol015404_RK

CTCTTTTCTGCGGGAGTATCTAATATATTAAAAGAGAAGTACACCTTTAAATTGCCGCTTAGTTTTGCAAGTTATTTACACTTCCATGCCACTAAGATATTAACTAAACCTATATTTAAGGATATTTTGTCTTTTACGGTTTAATTAATGCATTTGTTAAATTAATATATTTCTAATTAAAAACATTACAACATTAACATTCCACGGCTCCACTATCATATTCAACAACCAATACCTATTATTCCTCAATTCTTCAATTTATATACACAGCTTACTGAATCAAGCAAGGTTCCATTTCTCACCTCTTAGCTTATACAAATCGATTTTCTATCTTTTATAAACAAACAAAATCTCAATATACATCAAATCTACAATATTTATACATTTAGTTTTGTATTAATTCATACAAACAATACAAATCCATGTATCATATATCTATATCTATATATATATAATATTAACAACTATAAAATTTAATGATAATCAGTATACACAAATCGATTTTTCCAAAATCATAATGTAACTTAAGGATCAGACAGTTTTAAATTTCAAAATTAATGTATAAAATCTAACCAAAAAATAACACCTACTTAATTGCGATATTAGATCCTATTATATTTCTATATAAAATTTTATAATTAATGAGGAATATTTATTATAACAATAAGATTTGGAAACTAAAACACACAAAAACAATAATTTTCTTATTTGTTACTAAATCACATAATTAGTTACCACAATTATTCATTTCCCTTGACTTTCCCCTAGACTTGCGGATCAAACAACTTAAAATTTTAAAAAATTTAACGTATAAAATATATCCATAATTGACACTTTCTTACGGAATTTAGATCATAAATATCTACTATCAAACTATTTCCTATATTCTCGCTAACCAATATCACCTTTAATACAAACTTAAGCTTATTTATAATTTTTTATCAATTACAAAAAAATTACATTTTCATTTTATTTTCTTACAGATTTAAATCATAATTTTTATACGTTCCAAATTTAATAGAAATTTAACAATCATTTTATTTTCTTACAAATGTTATAACTAATGTACCATTTAAAAATAGCATACTAACTCATAATTGTCTATTTTCCATATTTTTCATTTTAAAAATTTGTTCGCTACAGATTTTTGACATGGATCTAATTATATAAATGTTGGATTTATACTCAATATTGGTAATACATTTTACGTTAAAACACATAAAAAAAGTACTCGATTTTATACAACTACATTTACAAATATAACATTATGTACAAACAAAAATTAAAATATAAAATTAAATACCCTTGCGGTCGCACGGGTTAAGATCTAGTTTTTCTTATTTATGAAGGCCATTTGTAATTATCTGTGGGAACAAGAAGTAGGACCTTAAATTTGTCATGTCAGAACGATAATATTGTGAAATGGAGCGTATAGAGTCGTAGAGAGGAAGGCGTTTTTTTCATACGGGGTCGAGATATTTTAAGGGTTTGATTTAGTCTTATTGTTAATTAGTTAATTACAATCACAAAAATATTATCCATCTCTTTAGATTTTTAAAAAATATATATAATAAATTAAAATTAAAAATTTAGATAGTATAAGTATAAATATAAATATCTTTGATATTTTCTAGTTGTATTCACTGATGACACTCATAATTCATATTTATAAGACTTTAAACCAAAAAATTTGTAGATCTTTTTTCTTAATCCCTAATATGCATATAGTACATACATAGGAATCTAATAAATTCATTTTTAAAATTTTAGTATCAATTTTTGACAAAAGAAAAAGTATTTCCATTAAATTTATAACTTTCATCTAAATAAAATAAAATAAATAAATAAATTTGCATGTTATTATTTATTATATAAATCAAGGATTTTATATTTCAGTATAAGAATATATTTTTAATATTTTATAATTATATAAAATGTGAATATATTATTAAATAATTCATGTATCTATTTTTCAACTAGTAATAATTATCCCGCAAATTCATTCGTCTTAACTAATGGGTTAGTATTATATGAATTTATTAACAACGATTAAAACCGTAACTATCCATATCTGTAAAATTTTACATCTGCACTCGCAACTGCTGCATTTAAACCAATTAAATCATACAAAGAATCATATTAATTCTACAGGCACAAAGTATTTTGATTTATCTTTTATATTTTCTGAATTTTAGTGATACTAAAAATACATTATCAGAACAAAGATAACATTATATTTCATCTTTATCAAACAGAAATCAAAAGTTGGAAATTTAAGAATGAAATATTGCTATTTATTGAAATGTGAAACTCTATTTTATTTCTATGTTAAAAGAACCTAAATAAGTGATTAACTATGTCTATGTTAGATTTTAAATATTACCAATTGATATAACTATGTCTATGTTAGATTTTTAATACCTTGTGACATTCTTCTATCCACGTAAGTAAATTTACTTTTTTCATTTTAGGATTCATCATCTATATATATGACCCACCAATGCATTGTTCAAGGCACAACAACATGAGCTTGCTTTGGATCACAGGATTGTGTGTTACAGCCCTAGTGGTTGTGAGGATTAGCAATTGGTGGTACCGATGGTCAAACCCCAAGTCTAATGGCATGTTACCTCCGGGATCAATGGGCTTCCCCATAATCGGAGAGACGTTCGATTTTTTTAAGCCGCATGGATTCTACGAGATCTCCCCGTTTCTCAAGAAGAAGATGTTAAGGTCAGGTTTCAATCATTTATATATGTGGATTCGATAAGCTGCTGTGGTTTAAGCTTTCCAATGGTTTGGATGTTGTTGGTCCATCTTGTTTTTATTCTTTTTTGCCAAAATGTATCAAACCTAAGATTTTGCTTTTGTAAAATCTTTTCTTCATTTATATGATATTTACAATTTAGCAAAAAAAAAATCATTTATATGTGTATATCATATTTCATTTCCTTAGAGATGAAATCTTGTATGTTTGTGTGTTGTGTGAGTGTTTCAGGTATGGACCTTTGTTTCGGACTAGCATTCTTGGTGTAAACACTGTAATTTCTACAGATATGGATGTGAACTTGGATATTTTACGACACGAGAACGAGTATTTTAATTTAAGTTATCCAGATGGTTTAGTGAAGCCATTGGGAAAAGAAAGCATGTTCTTGAAGACGGGAAACATCCACAAGCACATCAAAAAAATCTGTATGCGTCTTTTGGGCTCCGAGAATTTGAAGCGGAAGATAATAAAAGATATGGATCACGTGACATGCGAGCATCTTAATTTGAAGGCTGGCCAAAGAAGAGTCGACCTCAGGGACCTAGTTTCAAGTGTAATCTTTCTTTCTTTCTTTATTTTGTTTGTTGTCTTCTACAAATTTTGACTATAAATTTTTGAAGGCTATAAACAATTTACTAAAATAGTTTAATAATTTTTATTTTAAATTTGAAAATCTGTGTTTATGTAATATATTTTAAATTTTAGAAACCATGCATATGCCAATGTTTCATTTGCTTAGGTGTTAATGTTGTTGCGTGCTAGTTGACACTCTCTTATCCCTGTTTTGATTTTTGTAGTTGATAACAGCCCACTTGACACCAAAAGTGATAAGTGATCTCAAACCAGAGACTCAATCAATTCTTATGGAAAGCTTCAAAGCCTTCAGTTTTGATTGGTTTAGGACATCCTATATTCTCTCTGCGGGAAGGGGTCTCTACAAAACCCTTTGGGTGAGATTTATATATTTTTCATGGTTTTTCTCGGAGAATAAGTGATTTCTAATTTTCTATATATACATAATATTATACGTAGGCATGCAGACAGGGGATGAAGTTGATAAAGGATGTTTACACGAGTAGAATAACGTCAAGAGAGAAGTACAATGACTTCCTTGAGACAGCACTAGAAGAGTTAGAGAAAGAAGGAAGCTCAGTGAACGAAGATGTGATTGTAAGCCTCATCTTCACTCTTTCCGGTATCACTCAAGATACCACCTCTAAGGCCATTTGCATGGTTGTGAAATTCATATCGGAAAACCCAAAAGTTCTTGTAGAGTTGAAGGTACGTAGTAACATATGCATACTAACGGTGAAGCTAGTAAATTTTGCATCTAACCGAGTCTACTATATATAGCTTATTTGTCAAATGTGGCCTCATTTCAAATCTAATATAAGGCCTAAATTTAATGCTTTGTTGGTCTGATGGAAGAGCCGGCCTGGTGATAGTAGTCTAGTAGTTATGTTCCTATTAAATCAGCGCAGAAAGGTTTCCACTCATTGGCCAATGTAATGTAATTTGACAGAGAGAGCATGAGGCAATCCTTGCGAACAGAGAAGATAAAGAAGGCGGAGTTACTTGGGAAGAATTCAGACACAAGATGACTTTCACCAACATGGTAAGTTGATAACGTCACTTTGGTTTCATAAGGCCATATTGTACAGTATATTATCAGTTTATCACATTCACATGGTGTGTTTTTTGTTTACACACTCTAGGTTATACATGAGTCGCTTCGAATGACAAATTTGGCCCCTATGATGTTTAGAAAAGTGGTGAAAGATGTGGAAATAAAAGGCAAGTAGTATTGTACTAGTTTTACTATTATAATTAACAAACACAAAATAGTGTACTAATAATAATAAAATAATGTGGTACTAGACTACTAGTAATAAAATTGTATACAAAAAGCTGGACTTGATGATTAGTTGTTATTTGGCTTATACTTTTTATTTATTTCATATTCGCCTTGTATGACATTCAAGTTAAAAGTTTACAAATTTTTTTACTTAGAAAACGAATCAAATATCTAAATATTATTATATTTTTAATAATTGTCACATTACTTGCTCTATTATTTTTATTCATTATTCGTTAAAAAGATATATATTACTTCTTAAGAAATATTTGTTAGTCGTTATTTTTTTTTTTTTTTGCATTGTATTTTTTTTTAAAAAAATTGGATTAATAAGCAAAGTCCACTTATAAAAGGTAATAAAGCCTACACAAGAGAAAAAAAAATCCATTAAATTTCAAATCTTACAAGTAACTAGTGTCTATCCCTAGTATACATAGATTTTTGTTAAAAAGTTTCTTTTTGCTAACAGAATTTTTTGGTCCCAACAGGATATACGATTCCAGCAGATTGGATAGTGTTGGTTATACCTTCACTGGTTCATTTTGACCCTGAAATTTATGATAATCCATTTGAGTTTAATCCGTGGAGATGGGAGGTATTAACAGTTACTCTAGTCTATGTAAAAATAACATATATTATATTTATATTTTTCAAATCCTAAAGTTTTAATATATGATATTCTAGAATATATTTATATCCATCCTGATGATAAATATGATGGATAGGGGAAGGAGTTGCGGTCTGGGTCTAAAACGTTCATGGTGTTTGGAGCAGGAGCCAGACAGTGCGCTGGTTCAGAGTATGCAAGGCTTCAGATTTCACTGTTCATTCATCATCTTGTCACCACTTACGATTTCTCCTTATCCAAAGACTGCGAGGTGATTCGGGTACCAGGTGCTCTCTTCCCCAATGGCATCTCTATGAACATCTCCAAGCGTTCCAAGTGA

> Bol015404_WK

CTCTTTTCTGCGGGAGTATCTAATATATTAAAAGAGAAGTACACCTTTAAATTGCCGCTTAGTTTTGCAAGTTATTTACACTTCCATGCCACTAAGATATTAACTAAACCTATATTTAAGGATATTTTGTCTTTTACGGTTTAATTAATGCATTTGTTAAATTAATATATTTCTAATTAAAAACATTACAACATTAACATTCCACGGCTCCACTATCATATTCAACAACCAATACCTATTATTCCTCAATTCTTCAATTTATATACACAGCTTACTGAATCAAGCAAGGTTCCATTTCTCACCTCTTAGCTTATACAAATCGATTTTCTATCTTTTATAAACAAACAAAATCTCAATATACATCAAATCTACAATATTTATACATTTAGTTTTGTATTAATTCATACAAACAATACAAATCCATGTATCATATATCTATATCTATATATATATAATATTAACAACTATAAAATTTAATGATAATCAGTATACACAAATCGATTTTTCCAAAATCATAATGTAACTTAAGGATCAGACAGTTTTAAATTTCAAAATTAATGTATAAAATCTAACCAAAAAATAACACCTACTTAATTGCGATATTAGATCCTATTATATTTCTATATAAAATTTTATAATTAATGAGGAATATTTATTATAACAATAAGATTTGGAAACTAAAACACACAAAAACAATAATTTTCTTATTTGTTACTAAATCACATAATTAGTTACCACAATTATTCATTTCCCTTGACTTTCCCCTAGACTTGCGGATCAAACAACTTAAAATTTTAAAAAATTTAACGTATAAAATATATCCATAATTGACACTTTCTTACGGAATTTAGATCATAAATATCTACTATCAAACTATTTCCTATATTCTCGCTAACCAATATCACCTTTAATACAAACTTAAGCTTATTTATAATTTTTTATCAATTACAAAAAAATTACATTTTCATTTTATTTTCTTACAGATTTAAATCATAATTTTTATACGTTCCAAATTTAATAGAAATTTAACAATCATTTTATTTTCTTACAAATGTTATAACTAATGTACCATTTAAAAATAGCATACTAACTCATAATTGTCTATTTTCCATATTTTTCATTTTAAAAATTTGTTCGCTACAGATTTTTGACATGGATCTAATTATATAAATGTTGGATTTATACTCAATATTGGTAATACATTTTACGTTAAAACACATAAAAAAAGTACTCGATTTTATACAACTACATTTACAAATATAACATTATGTACAAACAAAAATTAAAATATAAAATTAAATACCCTTGCGGTCGCACGGGTTAAGATCTAGTTTTTCTTATTTATGAAGGCCATTTGTAATTATCTGTGGGAACAAGAAGTAGGACCTTAAATTTGTCATGTCAGAACGATAATATTGTGAAATGGAGCGTATAGAGTCGTAGAGAGGAAGGCGTTTTTTTCATACGGGGTCGAGATATTTTAAGGGTTTGATTTAGTCTTATTGTTAATTAGTTAATTACAATCACAAAAATATTATCCATCTCTTTAGATTTTTAAAAAATATATATAATAAATTAAAATTAAAAATTTAGATAGTATAAGTATAAATATAAATATCTTTGATATTTTCTAGTTGTATTCACTGATGACACTCATAATTCATATTTATAAGACTTTAAACCAAAAAATTTGTAGATCTTTTTTCTTAATCCCTAATATGCATATAGTACATACATAGGAATCTAATAAATTCATTTTTAAAATTTTAGTATCAATTTTTGACAAAAGAAAAAGTATTTCCATTAAATTTATAACTTTCATCTAAATAAAATAAAATAAATAAATAAATTTGCATGTTATTATTTATTATATAAATCAAGGATTTTATATTTCAGTATAAGAATATATTTTTAATATTTTATAATTATATAAAATGTGAATATATTATTAAATAATTCATGTATCTATTTTTCAACTAGTAATAATTATCCCGCAAATTCATTCGTCTTAACTAATGGGTTAGTATTATATGAATTTATTAACAACGATTAAAACCGTAACTATCCATATCTGTAAAATTTTACATCTGCACTCGCAACTGCTGCATTTAAACCAATTAAATCATACAAAGAATCATATTAATTCTACAGGCACAAAGTATTTTGATTTATCTTTTATATTTTCTGAATTTTAGTGATACTAAAAATACATTATCAGAACAAAGATAACATTATATTTCATCTTTATCAAACAGAAATCAAAAGTTGGAAATTTAAGAATGAAATATTGCTATTTATTGAAATGTGAAACTCTATTTTATTTCTATGTTAAAAGAACCTAAATAAGTGATTAACTATGTCTATGTTAGATTTTAAATATTACCAATTGATATAACTATGTCTATGTTAGATTTTTAATACCTTGTGACATTCTTCTATCCACGTAAGTAAATTTACTTTTTTCATTTTAGGATTCATCATCTATATATATGACCCACCAATGCATTGTTCAAGGCACAACAACATGAGCTTGCTTTGGATCACAGGATTGTGTGTTACAGCCCTAGTGGTTGTGAGGATTAGCAATTGGTGGTACCGATGGTCAAACCCCAAGTCTAATGGCATGTTACCTCCGGGATCAATGGGCTTCCCCATAATCGGAGAGACGTTCGATTTTTTTAAGCCGCATGGATTCTACGAGATCTCCCCGTTTCTCAAGAAGAAGATGTTAAGGTCAGGTTTCAATCATTTATATATGTGGATTCGATAAGCTGCTGTGGTTTAAGCTTTCCAATGGTTTGGATGTTGTTGGTCCATCTTGTTTTTATTCTTTTTTGCCAAAATGTATCAAACCTAAGATTTTGCTTTTGTAAAATCTTTTCTTCATTTATATGATATTTACAATTTAGCAAAAAAAAAATCATTTATATGTGTATATCATATTTCATTTCCTTAGAGATGAAATCTTGTATGTTTGTGTGTTGTGTGAGTGTTTCAGGTATGGACCTTTGTTTCGGACTAGCATTCTTGGTGTAAACACTGTAATTTCTACAGATATGGATGTGAACTTGGATATTTTACGACACGAGAACGAGTATTTTAATTTAAGTTATCCAGATGGTTTAGTGAAGCCATTGGGAAAAGAAAGCATGTTCTTGAAGACGGGAAACATCCACAAGCACATCAAAAAAATCTGTATGCGTCTTTTGGGCTCCGAGAATTTGAAGCGGAAGATAATAAAAGATATGGATCACGTGACATGCGAGCATCTTAATTTGAAGGCTGGCCAAAGAAGAGTCGACCTCAGGGACCTAGTTTCAAGTGTAATCTTTCTTTCTTTCTTTATTTTGTTTGTTGTCTTCTACAAATTTTGACTATAAATTTTTGAAGGCTATAAACAATTTACTAAAATAGTTTAATAATTTTTATTTTAAATTTGAAAATCTGTGTTTATGTAATATATTTTAAATTTTAGAAACCATGCATATGCCAATGTTTCATTTGCTTAGGTGTTAATGTTGTTGCGTGCTAGTTGACACTCTCTTATCCCTGTTTTGATTTTTGTAGTTGATAACAGCCCACTTGACACCAAAAGTGATAAGTGATCTCAAACCAGAGACTCAATCAATTCTTATGGAAAGCTTCAAAGCCTTCAGTTTTGATTGGTTTAGGACATCCTATATTCTCTCTGCGGGAAGGGGTCTCTACAAAACCCTTTGGGTGAGATTTATATATTTTTCATGGTTTTTCTCGGAGAATAAGTGATTTCTAATTTTCTATATATACATAATATTATACGTAGGCATGCAGACAGGGGATGAAGTTGATAAAGGATGTTTACACGAGTAGAATAACGTCAAGAGAGAAGTACAATGACTTCCTTGAGACAGCACTAGAAGAGTTAGAGAAAGAAGGAAGCTCAGTGAACGAAGATGTGATTGTAAGCCTCATCTTCACTCTTTCCGGTATCACTCAAGATACCACCTCTAAGGCCATTTGCATGGTTGTGAAATTCATATCGGAAAACCCAAAAGTTCTTGTAGAGTTGAAGGTACGTAGTAACATATGCATACTAACGGTGAAGCTAGTAAATTTTGCATCTAACCGAGTCTACTATATATAGCTTATTTGTCAAATGTGGCCTCATTTCAAATCTAATATAAGGCCTAAATTTAATGCTTTGTTGGTCTGATGGAAGAGCCGGCCTGGTGATAGTAGTCTAGTAGTTATGTTCCTATTAAATCAGCGCAGAAAGGTTTCCACTCATTGGCCAATGTAATGTAATTTGACAGAGAGAGCATGAGGCAATCCTTGCGAACAGAGAAGATAAAGAAGGCGGAGTTACTTGGGAAGAATTCAGACACAAGATGACTTTCACCAACATGGTAAGTTGATAACGTCACTTTGGTTTCATAAGGCCATATTGTACAGTATATTATCAGTTTATCACATTCACATGGTGTGTTTTTTGTTTACACACTCTAGGTTATACATGAGTCGCTTCGAATGACAAATTTGGCCCCTATGATGTTTAGAAAAGTGGTGAAAGATGTGGAAATAAAAGGCAAGTAGTATTGTACTAGTTTTACTATTATAATTAACAAACACAAAATAGTGTACTAATAATAATAAAATAATGTGGTACTAGACTACTAGTAATAAAATTGTATACAAAAAGCTGGACTTGATGATTAGTTGTTATTTGGCTTATACTTTTTATTTATTTCATATTCGCCTTGTATGACATTCAAGTTAAAAGTTTACAAATTTTTTTACTTAGAAAACGAATCAAATATCTAAATATTATTATATTTTTAATAATTGTCACATTACTTGCTCTATTATTTTTATTCATTATTCGTTAAAAAGATATATATTACTTCTTAAGAAATATTTGTTAGTCGTTATTTTTTTTTTTTTTTGCATTGTATTTTTTTTTAAAAAAATTGGATTAATAAGCAAAGTCCACTTATAAAAGGTAATAAAGCCTACACAAGAGAAAAAAAAATCCATTAAATTTCAAATCTTACAAGTAACTAGTGTCTATCCCTAGTATACATAGATTTTTGTTAAAAAGTTTCTTTTTGCTAACAGAATTTTTTGGTCCCAACAGGATATACGATTCCAGCAGATTGGATAGTGTTGGTTATACCTTCACTGGTTCATTTTGACCCTGAAATTTATGATAATCCATTTGAGTTTAATCCGTGGAGATGGGAGGTATTAACAGTTACTCTAGTCTATGTAAAAATAACATATATTATATTTATATTTTTCAAATCCTAAAGTTTTAATATATGATATTCTAGAATATATTTATATCCATCCTGATGATAAATATGATGGATAGGGGAAGGAGTTGCGGTCTGGGTCTAAAACGTTCATGGTGTTTGGAGCAGGAGCCAGACAGTGCGCTGGTTCAGAGTATGCAAGGCTTCAGATTTCACTGTTCATTCATCATCTTGTCACCACTTACGATTTCTCCTTATCCAAAGACTGCGAGGTGATTCGGGTACCAGGTGCTCTCTTCCCCAATGGCATCTCTATGAACATCTCCAAGCGTTCCAAGTGA

> Bol015404_GC

CGGTAGTATCTAATATATTAAAAGAGAAGTACACCTTTAAATTACCGCTTAGTTTTGCAAGTTATTTACACTTCCATGCCACTAAGATATTAACTAAACCTATATTTAAGGATATTTTGTCTTTTACGGTTTAATTAATGCATTTGTTAAATTAATATATTTCTAATTAAAAACATTACAACATTAACATTCCACGGCTCCACTATCATATTCAACAACCAATACCTATTATTCCTCAATTCTTCAATTAATATACACAGCTTACTGAATCAAGCAAGGTTCCATTTCTCACCTCTTAGCTTATACAAATCGATTTTCTATCTTTTATAAACAAACAAAATCTCAATATACATCAAATCTACAATATTTATACATTTAGTTTTGTATTAATTCATACAAACAATACAAATCCATGTATCATATATCTATATCTATATCTATATCTATATATATAATATTAACAACTATAAAATTTAATGATAATCAGTATACACAAATCGATTTTTCCAAAATCATAATGTAACTTAAGGATCAGACAGTTTTAAATTTCAAAATTAATGTATAAAATCGAACCAAAAAATAACACCTACTTAATTGCGATATTAGATCCTATTATATTTCTATATAAAATTTTATAATTAATGAGGAATATTTACTATAACAAAAAGATTTGGAAACTAAAACACACAAAAACAATAATTTTCTTATTTGTTACTAAATCACATAATTAGTTACCACAATTATTCATTTCCCTTGACTTTCCCCTAGACTTGCGGATCAAACAACTTAAAATTTTAAAAAATTTAACGTATAAAATATATCCATAATTGACACTTTCTTACGAAATTTAGATCATAAATATCTACTATCAAACTATTTCCTATATTCTCGCTAACCAATATCACCTTTAATACAAACTTAAGCTTATTTATAATTTTTTATCAATTACAAAAAAATTACATTTTCATTTTATTTTCTTACAGATTTAAATCATAATTTTTATACGTTCCAAATTTAATAGAAATTTAACAATCATTTTATTTTCTTACAAATGTTATAACTAATGTACCATTTAAAAATAGCATACTAACTCATAATTGTCTATTTTCCATATTTTTCATTTTAAAAATTTGTTCGCTACAGATTTTTGACATGGATCTAATTATATAAATGTTGGATTTATACTCAATATTGGTAATACATTTTACGTTAAAACACATAAAAAAAGTACTCGATTTTATACAACTACATTTACAAATATAACATTATGTACAAACAAAAATTAAAATATAAAATTAAATACCCTTGCAGTCGCACGGGTTAAGATCTAGTTTTTCTTATTTATGAAGGCCATTTGTAATTATCTGTGGGAACAAGAAGTAGGACCTTAAATTTGTCATGTCAGAACGATAATATTGTGAAATGGAGCGTATAGAGTCGTGGAGAGGAAGGCGTTTTTTTCATACGGGGTCGAGATATTTTAAGGGTTTGATTTAGTCTTATTGTTAATTAGTTAATTACAATCACAAAAATATTATCCATCTCTTTAGATTTTTAAAAAATATATATAATAAATTAAAATTAAAAATTTAGATAGTATAAGTATAAATATAAATATCTTTGATATTTTCTAGTTGTATTCACTGATGACACTCATAATTCATATTTATAAGACTTTAAACCAAAAAAAATGTAGATCTTTTTTCTTAATCCCTAATATGCATATAGTACATACATAGGAATCTAATAAATTCATTTTTAAAATTTTAGTATCAATTTTTGACAAAAGAAAAAGTATTTCCATTAAATTTATAACTTTCATCTAAATAAAATAAAATAAATAAATAAATTTGCATGTTATTATTTATTATATAAATCAAGGATTTTATATTTCAGTATAAGAATATATTTTTAATATTTTATAATTATATAAAATGTGAATATATTATTAAATAATTCATGTATCTATTTTTCAACTAGTAATAATTATCCCGCAAATTCATTCGTCTTAACTAATGGGTTAGTATTATATGAATTTATTAACAACGATTAAAACCGTAACTATCCATATCTGTAAAATTTTACATCTGCACTCGCAACTGCTGCATTTAAACCAATTAAATCATACAAAGAATCATATTAATTCTACAGGCACAAAGTATTTTGATTTATCTTTTATATTTTCTGAATTTTAGTGATACTAAAAATACATTATCAGAACAAAGATAACATTATATTTCATCTTTATCAAACAGAAATCAAAAGTTGGAAATTTAAGAATGAAATATTGCTATTTATTGAAATGTGAAACTCTATTTTATTTCTATGTTAAAAGAACCTAAATAAGTGATTAACTATGTCTATGTTAGATTTTAAATATTACCAATTGATATAACTATGTCTATGTTAAATTTTTAATACCTTGTGACATTCTTCTATCCACGTAAGTAAATTTACTTTTTTCATTTTAGGATTCATCATCTATATATATGACCCACCAATGCATTGTTCAAGGCACAACAACATGAGCTTGCTTTGGATCACAGGATTGTGTGTTACAGCCCTAGTGGTTGTGAGGATTAGCAATTGGTGGTACCGATGGTCAAACCCCAAGTCTAATGGCATGTTACCTCCGGGATCAATGGGCTTCCCCATAATCGGAGAGACGTTCGATTTTTTTAAGCCGCATGGATTCTACGAGATCTCCCCGTTTCTCAAGAAGAAGATGTTAAGGTCAGGTTTCAATCATTTATATATGTGGATTCGATAAGCTGCTGTGGTTTAAGCTTTCCAATGGTTTGGATGTTGTTGGTCCATCTTGTTTTTATTCTTTTTTGCCAAAATGTATCAAACCTAAGATTTTGCTTTTGTAAAATATTTTCTTCATTTATATGATATTTACAATTTAGCAAAAAAAAAATCATTTATATATGTGTATATCATATTTCATTTCCTTAGAGATGAAATCTTGTATGTTTGTGTGTTGTGTGAGTGTTTCAGGTATGGACCTTTGTTTCGGACTAGCATTCTTGGTGTAAACACTGTAATTTCTACAGATATGGATGTGAACTTGGATATTTTACGACACGAGAACGAGTATTTTAATTTAAGTTATCCAGATGGTTTAGTGAAGCCATTGGGAAAAGAAAGCATGTTCTTGAAGACGGGAAACATCCACAAGCACATCAAAAAAATCTGTATGCGTCTTTTGGGCTCCGAGAATTTGAAGCGGAAGATAATAAAAGATATGGATCACGTGACATGCGAGCATCTTAATTTGAAGGCTGGCCAAAGAAGAGTCGACCTCAGGGACCTAGTTTCAAGTGTAATCTTTCTTTCTTTCTTTATTTTGTTTGTTGTCTTCTACAAATTTTGACTATAAATTTTTGAAGGCTATAAACAATTTACTAAAATAGTTTAATAATTTTTATTTTAAATTTGAAAATCTGTGTTTATGTAATATATTTTCAATTTTAGAAACCATGCATATGCCAATGTTTCATTTGCTTAGGTGTTAATGTTGTTGCGTGCTAGTTGACACTCTCTTATCCCTGTTTTGATTTTTGTAGTTGATAACAGCCCACTTGACACCAAAAGTGATAAGTGATCTCAAACCAGAGACTCAATCAATTCTTATGGAAAGCTTCAAAGCCTTCAGTTTTGATTGGTTTAGGACATCCTATATTCTCTCTGCGGGAAGGGGTCTCTACAAAACCCTTTGGGTGAGATTTATATATTTTTCATGGTTTTTCTCGGAGAATAAGTGATTTCTAATTTTCTATATATACATAATATTATACGTAGGCATGCAGACAGGGGATGAAGTTGATAAAGGATGTTTACACGAGTAGAATAACGTCAAGAGAGAAGTACAATGACTTCCTTGAGACAGCACTAGAAGAGTTAGAGAAAGAAGGAAGCTCAGTGAACGAAGATGTGATTGTAAGCCTCATCTTCACTCTTTCCGGTATCACTCAAGATACCACCTCTAAGGCCATTTGCATGGTTGTGAAATTCATATCGGAAAACCCAAAAGTTCTTGTAGAGTTGAAGGTACGTAGTAACATATGCATACTAACGGTGAAGCTAGTAAATTTTGCATCTAACCGAGTCTACTATATATAGCTTATTTGTCAAATGTGGCCTCATTTCAAATCTAATATAAGGCCTAAATTTAATGCTTTGTTGGTCTGATGGAAGAGCCGGCCTGGTGATAGTAGTCTAGTAGTTATGTTCCTATTAAATCAGCGCAGAAAGGTTTCCACTCATTGGCCAATGTAATGTAATTTGACAGAGAGAGCATGAGGCAATCCTTGCGAACAGAGAAGATAAAGAAGGCGGAGTTACTTGGGAAGAATTCAGACACAAGATGACTTTCACCAACATGGTAAGTTGATAACGTCACTTTGGTTTCATAAGGCCATATTGTACAGTATATTATCAGTTTATCACATTCACATGGTGTGTTTTTTGTTTACACACTCTAGGTTATACATGAGTCGCTTCGAATGACAAATTTGGCCCCTATGATGTTTAGAAAAGTGGTGAAAGATGTGGAAATAAAAGGCAAGTAGTATTGTACTAGTTTTACTATTATAATTAACAAACACAAAATAGTGTACTAATAATAATAAAATAATGTGGTACTAGTAATAAAATTGTATACAAAAAGCTGGACTTGATGATTAGTTGTTATTTGGCTTATACTTTTTATTTATTTCATATTCGCCTTGTATGACATTCAAGTTAAAAGTTTACAAATTTTTTTACTTAGAAAACGAATCAAATATCTAAATATTATTATATTTTTAATAATTGTCACATTACTTGCTCTATTATTTTTATTCATTATTCGTTAAAAAGATATATATTACTTCTTAAGAAATATTTGTTAGTCGTTATTTATTTTTTTTTTTGCATTGTATTTTTTTTAAAAAAAATTGGATTAATAAGCAAAGTCCACTTATAAAAGGTAATAAAGCCTACACAAGAGAAAAAAAAATCCATTAAATTTCAAATCTTACAAGTAACTAGTGTCTATCCCTAGTATACATAGATTTTTGTTAAAAAGTTTCTTTTTGCTAACAGAATTTTTTGGTCCCAACAGGATATACGATTCCAGCAGATTGGATAGTGTTGGTTATACCTTCACTGGTTCATTTTGACCCTGAAATTTATGATAATCCATTTGAGTTTAATCCGTGGAGATGGGAGGTATTAACAGTTACTCTAGTCTATGTAAAAATAACATATATTATATTTATATTTTTCAAATCCTAAAGTTTTAATATATGATATTCTAGAATATATTTATATCCATCCTGATGATAAATATGATGGATAGGGGAAGGAGTTGCGGTCTGGGTCTAAAACGTTCATGGTGTTTGGAGCAGGAGCCAGACAGTGCGCTGGTTCAGAGTATGCAAGGCTTCAGATTTCACTGTTCATTCATCATCTTGTCACCACTTACGATTTCTCCTTATCCAAAGACTGCGAGGTGATTCGGGTACCAGGTGCTCTCTTCCCCAATGGCATCTCTATGAACATCTCCAAGCGTTCCAAGTGA
